# Turing-like mechanism in a stochastic reaction-diffusion model recreates three dimensional vascular patterning of plant stems

**DOI:** 10.1371/journal.pone.0219055

**Published:** 2019-07-24

**Authors:** David J. Hearn

**Affiliations:** Department of Biological Sciences, Towson University, Towson, Maryland, United States of America; Max Planck Institute for Plant Breeding Research, CANADA

## Abstract

Vascular tissue in plants provides a resource distribution network for water and nutrients that exhibits remarkable diversity in patterning among different species. In many succulent plants, the vascular network includes longitudinally-oriented supplemental vascular bundles (SVBs) in the central core of the succulent stems and roots in addition to the more typical zone of vascular tissue development (vascular cambium) in a cylinder at the periphery of the succulent organ. Plant SVBs evolved in over 38 plant families often in tandem with evolutionary increases in stem and root parenchyma storage tissue, so it is of interest to understand the evolutionary-developmental processes responsible for their recurrent evolution and patterning. Previous mathematical models have successfully recreated the two-dimensional vascular patterns in stem and root cross sections, but such models have yet to recreate three-dimensional vascular patterning. Here, a stochastic reaction-diffusion model of plant vascular bundle patterning is developed in an effort to highlight a potential mechanism of three dimensional patterning–Turing pattern formation coupled with longitudinal efflux of a regulatory molecule. A relatively simple model of four or five molecules recreated empirical SVB patterns and many other common vascular arrangements. SVBs failed to develop below a threshold width of parenchymatous tissues, suggesting a mechanism of evolutionary character loss due to changes in the spatial context in which development takes place. Altered diffusion rates of the modeled activator and substrate molecules affected the number and size of the simulated SVBs. This work provides a first mathematical model employing a stochastic Turing-type mechanism that recreates three dimensional vascular patterns seen in plant stems. The model offers predictions that can be tested using molecular-genetic approaches. Evolutionary-developmental ramifications concerning evolution of diffusion rates, organ size and geometry are discussed.

## Introduction

The diversity of biological patterning is astonishing. Underlying much of that diversity are conserved genetic modules whose functioning, number, and timing, rate, and location of activity vary from lineage to lineage [1]. Vascular tissues in plants present one suite of biological patterns that function as resource distribution networks that carry water and nutrients throughout the plant body and simultaneously provide mechanical support [2]. The evolution of vascular tissues represents one of the most important adaptations of land plants. Vascular tissues' functions of conduction and support permitted the invasion of otherwise inaccessible arid, terrestrial regions and contributed to vascular plant long distance dispersal and competitive dominance [2,3].

Interest in modeling vascular development has grown recently, and multiple models stemming from a reaction-diffusion framework or molecular pathway analysis have recreated the various arrangements of vascular bundles in plant stems and roots. The classic pattern consists of a cylinder of vascular tissue or radially-arranged vascular bundles near the periphery of stems and roots [4–12] with water conducting tissue (xylem) developing proximally to the zone of vascular differentiation (the vascular cambium) and carbohydrate-transporting tissue (phloem) developing distally.

Thus far, mathematical models have not recreated three dimensional patterning that is essential for resource distribution, and many of these models do not explore patterning due to variant cambial activities that alter vascular patterning from the classic pattern. Here, a model is developed to illustrate potential mechanisms of evolution and development of both classic vascular arrangements in stems as well as vascular patterning of supplemental vascular bundles (SVBs) in ground tissue of stems and roots.

Previous modeling work presents common themes that underlie patterning processes in biological systems. In animals, models provided insights about mechanisms of developmental patterning of hair [13], reptile scales [14], feathers [15], and spotting and striping in multiple taxa [16–20]. In plants, mechanisms responsible for the arrangement of leaves on stems (phyllotaxis) [21,22] and the patterning of hairs (trichomes) on leaves [23,24] are also understood at some level. Although it is unlikely that a single mechanism is responsible for the patterning of such repeating, serially homologous units, these models consist of common components: reactions among the molecules, movement of molecules, and boundaries that define limits to the movement of the molecules. Often such models include an activator molecule that initiates development of a serially homologous feature (e.g., hair, trichome, leaf primordium) when it reaches a sufficiently high concentration. Many of the models also include an inhibitor molecule whose production is fostered by the activator and which suppresses both the activator and the development of the feature in adjacent regions.

In 1952, Alan Turing [25] encapsulated these core dynamics in the reaction-diffusion framework. This framework models both the movement of molecules as well as their reaction dynamics using (mathematically) continuous partial differential equations (PDEs). Munteanu and Solé [26] refer to this continuous approach of reaction-diffusion modelling as "the standard tool for evo-devo". Reaction-diffusion models cast evo-devo models in a predictive framework [15], as the developmental, and hence phenotypic, impacts due to evolutionary changes in gene expression rates, molecular degradation rates, diffusion rates, or reaction kinetics can be predicted directly from an analysis (or simulation) of the reaction-diffusion models.

**Fig 1:**
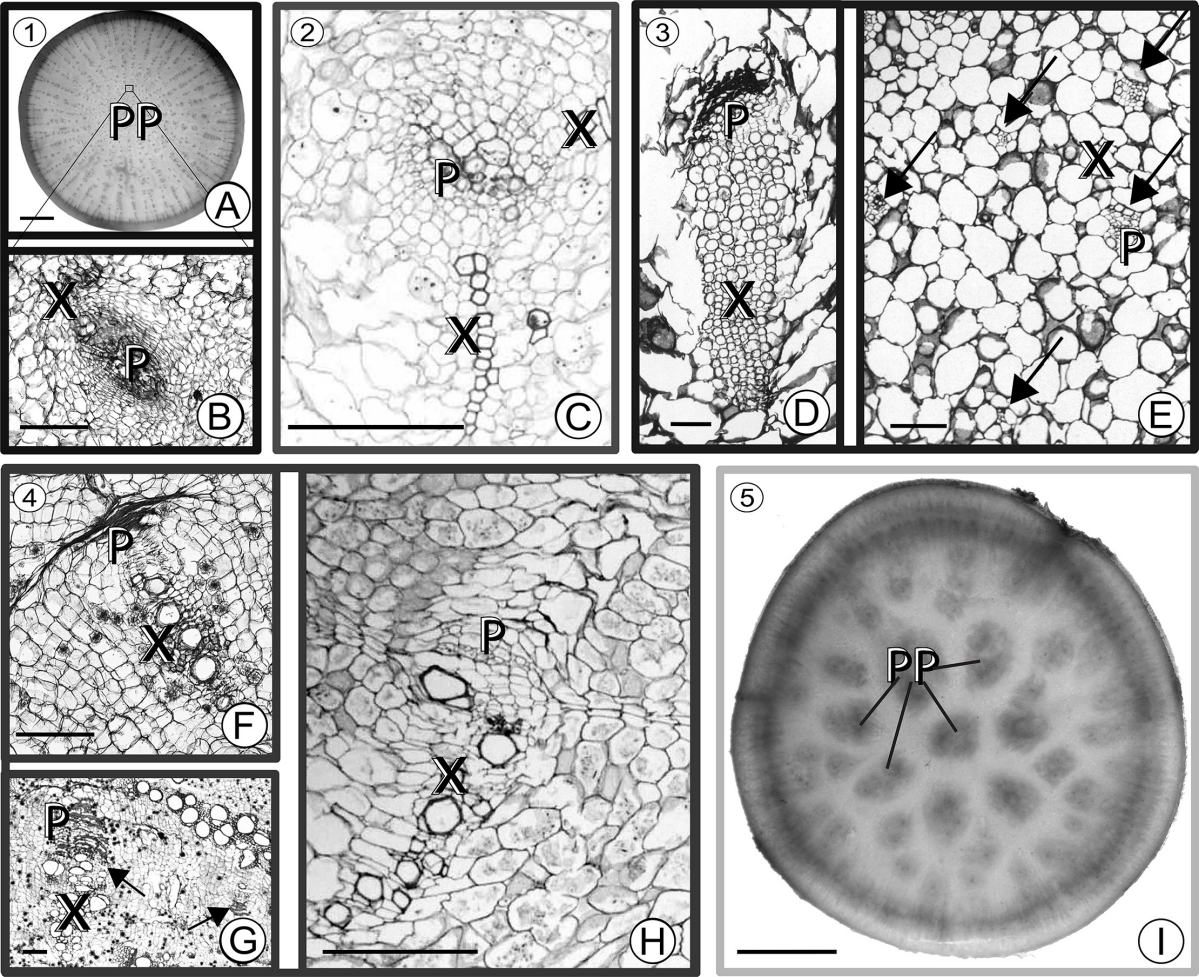
Multiple evolutionary origins of supplemental vascular bundles in stems and roots. (A) Turnip [*Brassica rapa rapa* (Brassicaceae)] tuberous root cross section. Small dots in the center of the section are vascular bundles in a zone where parenchyma cells are proliferating (PP). (B) Close-up of vascular bundle in turnip root tuber. A small zone of xylem (X) encircles crushed internal phloem (P) in the amphivasal bundle arrangement. (C) Older medullary SVB (i.e., SVB in pith) from stem of *Pachypodium namaquanum* (Apocynaceae) with two zones of xylem and a zone of phloem interior to the xylem. (D) Close-up of an old medullary SVB from the trunk of *Trichocereus chilensis* (Cactaceae) showing a long tail of xylem and phloem that is capped with crushed secondary phloem (dark band). (E) Cross section of young stem just below the shoot apical meristem of *Subpilocereus ottoni* (Cactaceae) showing the distribution of five medullary SVBs (arrows). (F) Old collateral vascular bundle from root tuber of *Adenia inermis* (Passifloraceae). (G) Large (note scale) collateral medullary SVBs from stem of *Adenia metamorpha*. (H) Old collateral medullary SVB from stem of *Adenia keramanthus*. (I) Cross section through tuberous root of *Ipomoea batatas* (Convolvulaceae). Dark spots in the center of the root are zones of proliferating parenchyma and vascular bundles. Taxonomic orders (numbered blocks) 1 –Brassicales; 2 –Caryophyllales; 3 –Gentianales; 4 –Malpighiales; 5 –Solanales. Scale: (A), (J)– 10mm; (B)-(H)– 250 μm. (C) adapted from [29] with permission; (D), (E) adapted from [32] with permission.

Patterning of supplemental vascular bundles (SVBs) raises interesting developmental and evolutionary questions. SVBs evolved in at least 38 plant families (Fig 1) [27–31] and occur in both stem and root tubers [30]. Like other vascular bundles, SVBs have vascular cambia that produce xylem and phloem in various configurations, but SVBs are separate from the secondary vascular cambium that occurs in a cylinder at the periphery of stems and roots of wood-forming plants. In contrast, SVBs tend to occur in the centers of succulent stems and roots or in wide stem cortices of plants such as cacti (Fig 1). They tend to evolve in tandem with evolutionary increases in parenchyma storage tissues [30] and are implicated in distributing resources within these putatively diffusion-limited storage structures [28,30,33]. As such, regions of proliferating storage parenchyma and SVBs are often associated with xeric succulent life forms such as cacti, as well as with plants whose tubers are of agronomic importance such as turnips, kohlrabi, and sweet potatoes.

**Fig 2:**
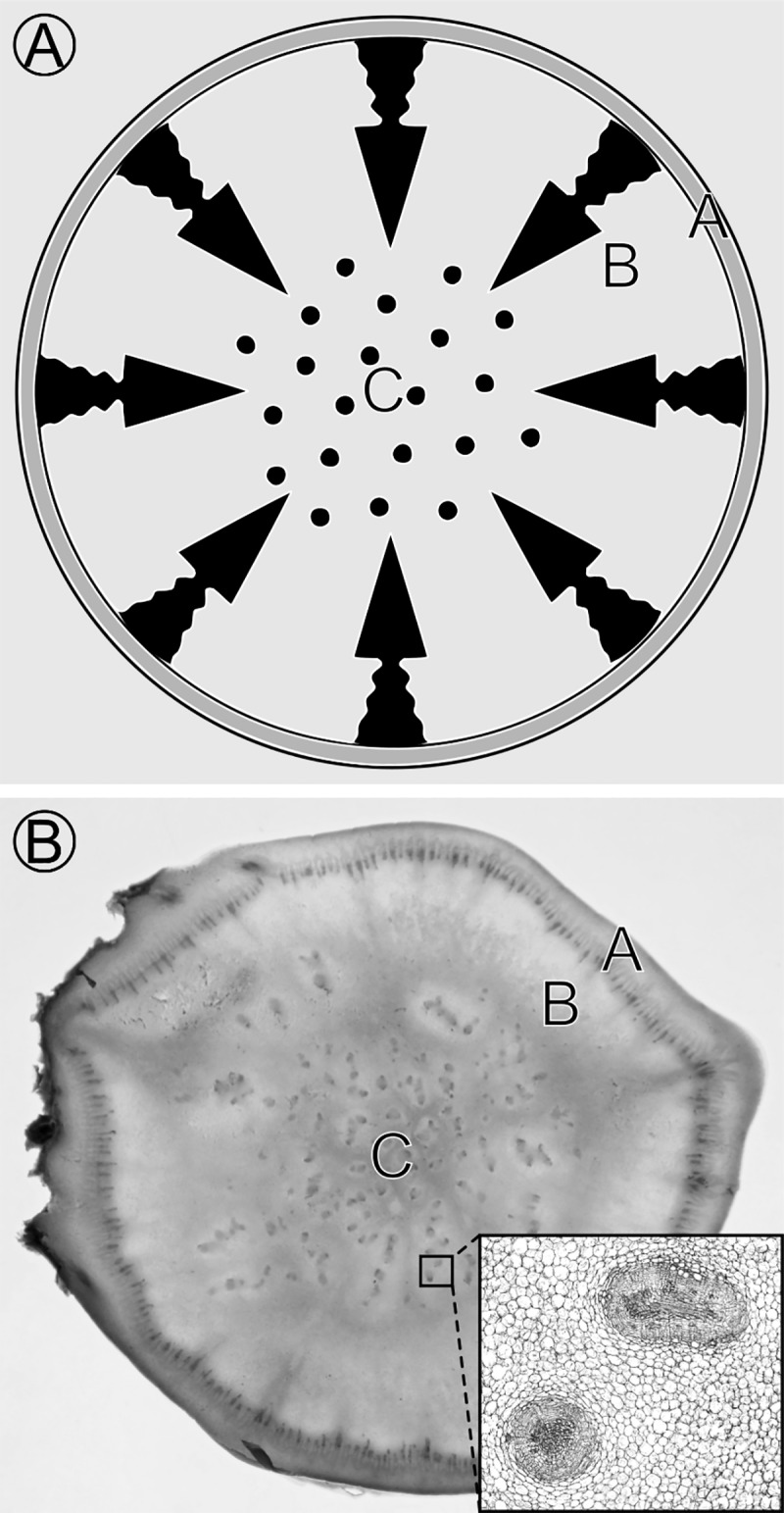
Spatial context of the reaction-diffusion model in 2D with molecules *H*, *B*, and *M*. (A) *H* (regulatory molecule responsible for vascular development initiation) and *B* (a substrate of *H*) are constitutively produced everywhere, whereas production of an inhibitory molecule, *M*, is restricted to the cortex in this model. *M* diffuses (arrows) from regions ‘A’ (cortex) into ‘B’ and suppresses SVB development by reducing the level of *H*. Upon release of suppression in region ‘C’ (medulla or pith), *H*, in concert with *B*, promotes differentiation of SVBs (black spots). (B) Cross section through a wide kohlrabi (*Brassica oleracea gongylodes*) stem provides an example that follows the ‘A’ ‘B’ ‘C’ zonation and SVB patterning of (A). Inset shows close-up of two SVBs. Stem diameter is ~6.5 cm. Stem regions ‘A’ = cortex, ‘B’ = stem region with no SVBs next to vascular cambium (inner black circle), ‘C’ = pith stem region with SVBs.

Within parenchymatous roots and shoots, SVBs are dispersed within the cortices, pith, or, more generally, centers of stems and roots, forming a spotted pattern in cross section and a matrix of (usually) longitudinally-oriented vessels in longitudinal section. In particular, four features of this network of SVBs are distinctive. First, these SVBs are often localized in the pith with zones of parenchyma separating them from the secondary vascular cambium (Fig 2 region B). Second, they tend to have a fairly uniform pattern of dispersion but deviate from complete uniformity. Third, they appear to be relatively rare in thin stems and roots, but more frequent in wide succulent stems, hypocotyls, or roots such as those in *Adenia* [30,34,35], Cactaceae [32,33], and *Brassica* (SVBs in turnip roots and kohlrabi stems illustrated in Figs 1 and 2 are absent from thin-stemmed kale and bak choi [36]), and they show a pattern of repeated evolutionary gain and loss in some clades [30]. Fourth, the number, size, density, and spatial distribution of regions differentiating into SVBs vary from taxon to taxon (compare turnip to sweet potato in Fig 1).

A stochastic, reaction-diffusion model consisting of interacting molecules (labeled *H*, *B*, *M*, *P*, and *A*) was developed to highlight potential mechanisms of three dimensional vascular patterning. To address the hypothesis that changes in stem and root size alone can cause changes in developmental patterning of SVBs, simulations investigated varying widths of stems. SVBs were expected to develop in wide stems and expected not to develop in narrow stems even when the molecular networks driving pattern formation are identical in wide and narrow stems. To investigate the role that changes in molecular diffusion rates have on patterning, diffusion rates of a regulatory molecule (labeled *H*) and its associated substrate (*B*) were varied while all other model parameters were kept constant. Application of Wei and Winter’s theory [37] predicted the number and size of SVBs in 2D sections. Their work predicted that the ratio of the diffusion rates of substrate and regulatory molecules most directly influence the density of SVBs, whereas the magnitude of the diffusion rate of the regulatory molecule influences their spatial scale. By altering additional parameters in the model that is developed here, other vascular patterns from a diversity of vascular plant lineages were recreated. Results of these simulations are discussed in terms of potential evolutionary-developmental mechanisms and their connections to known molecular regulators of vascular development.

## Methods

### Model creation

#### The core 2D model

To derive a reaction-diffusion model of vascular bundle patterning, a “core” model of two reacting components formed a foundation that was augmented, molecule by molecule, to arrive at a four-molecule *HBPM* model. Ultimately, this model needed additional augmentation with a fifth molecule, *A*, to correspond better with previously observed biological processes. In this methods section, outcomes of the simpler models and their limitations are described in order to motivate the more complex models.

Gray and Scott [38] and Schnakenberg [39] introduced a set of reaction diffusion models that form the core of the current model. These previous models are recognized for the diversity of spotted and striped patterns that they produce in 2D and 3D spaces. Both the Gray-Scott and Schnakenberg models have the same core activator-substrate dynamics with two interacting components. In the model, a dimer, here labeled *H*, autocatalyzes its formation in the presence of a ‘substrate’ (labeled *B*) that is consumed when it interacts with *H*. (The [*H*]^2^ term in Eqs 1 and 2 below is interpretable as a bi-molecular, or dimeric, interaction.). A Gray-Scott-Schnakenberg system of equations describing activator-substrate temporal and spatial dynamics is:

$$\frac{\partial [H]}{\partial t}=D_{H}\nabla^{2}[H]+{k_{1}[H]}^{2}[B]+k_{4}-k_{5}[H]$$ (Eq 1)

$$\frac{\partial [B]}{\partial t}=D_{B}\nabla^{2}[B]-{k_{1}[H]}^{2}[B]+k_{2}-k_{3}[B]$$ (Eq 2)

The *k*_*i*_ values are rate constants, the *D*_*i*_ values are diffusion rates, and the ∇^2^ Laplacian diffusion operator determines rates of movement between (spatial) regions.

Eqs 1 and 2 model the concentrations of *H* and *B* as a deterministic system of non-negative, real-valued numbers that can take arbitrarily small positive values. When considering molecules interacting in cells, this framework may not be appropriate, as the number of molecules is whole number-valued, and stochasticity is an intrinsic part of molecular dynamics in biological systems [40], especially when low numbers of molecules (seen in many regulatory molecules) are present in the cell. For example, under some parameterizations, the Schnakenberg model creates ‘stochastic resonance’ in which spurts of chemical concentrations occur when modeled as a stochastic process, whereas the deterministic model admits a constant steady state; additionally, reaction trajectories can shift between locally stable states in stochastic systems (e.g., the Schlogl model [41]). These stochastic outcomes are implemented as examples in the software developed herein to model vascular evo-devo (S1 Program).

To accommodate the possibility of stochastic effects, the “core” *HB* model (Eqs 1 and 2) developed here tracked each molecule as it diffused and reacted stochastically according to the above chemical reactions, a departure from previous models of vascular development that used continuous partial differential equations. To implement the stochastic model, the PDE of Eqs 1 and 2 was represented as a set of chemical reactions (Reactions *X*_*1*_ –*X*_*5*_ of Table 1). This framework using chemical reaction formulae to model chemical dynamics was illustrated in Turing’s 1952 paper [25] and further developed by Gillespie [42]. An exact stochastic simulation using the stochastic simulation algorithm (SSA) of Gillespie [42] produces a realization of the stochastic master equation that models the chemical reactions between *B* and *H*.

**Table 1:** Chemical reactions.

Reaction Label	Chemical Reaction	Description	Reaction Rate
*HB X*_*1*_	2*H*+*B*→3*H*	Core reaction of Gray-Scott [38] and Schnakenberg [39] models	*k*_*1*_
*HB X*_*2*_	Ø*→B*	Production of *B*	*k*_*2*_
*HB X*_*3*_	*B*→Ø	Degradation of *B*	*k*_*3*_
*HB X*_*4*_	Ø*→H*	Production of *H*	*k*_*4*_
*HB X*_*5*_	*H*→Ø	Degradation of *H*	*k*_*5*_
*HBP X*_*6*_	*H*→*H*+*P*	Production of carrier, *P*	*k*_*6*_
*HBP X*_*7*_	*P→*Ø	Degradation of *P*	*k*_*7*_
*HBP X*_*8*_	*P*↑↓*H*	Indirect longitudinal transport of *H*	*α*
*HBPM X*_*9*_	Ø*→M*	Production of *M*	*k*_*9*_
*HBPM X*_*10*_	*M*→Ø	Degradation of *M*	*k*_*10*_
*HBPM X*_*11*_	*M*+*H*→Ø	*M* and *H* annihilate each other	*k*_*11*_
*HBPM X*_*12*_	*M*+*B*→Ø	*M* and *B* annihilate each other in a subset of model iterations to remove peripheral vascular bundles and focus on medullary SVBs.	*k*_*12*_

Notation and interpretation of reactions are provided. Reaction *X*_*9*_ is constrained to the outer 4% of the cylindrical shell of the simulated stem. All other reactions occur throughout the simulated stem. Diffusion rates for *H*, *B*, and *M* are *D*_*H*_, *D*_*B*_, and *D*_*M*_, respectively. Diffusion rate for *P* was 0. *X*↑↓*Y* indicates that molecule *X* facilitates transport of *Y* longitudinally. See text below for further description.

Relatively recent advances in Monte Carlo sampling make this simulation approach feasible for a large number of reactions [43,44]. S1 Appendix provides details concerning algorithms and implementation. As S1 Appendix describes, all simulations took place in a discretized lattice of equal-volume cubical regions, hereafter called “voxels”. This finite element approach is a standard approach to model spatial processes, and a similar finite element approach was used previously to model plant vascular patterning (e.g., [6]). Simulations were carried out in either a circle in 2D (representing a stem cross section) or in a right circular cylindrical solid in 3D (representing a plant stem), and boundaries of the circle or cylinder were reflective (zero-flux Neumann boundary conditions). Reactants were initialized to concentrations of 0.

The reaction-diffusion SSA was implemented in a Java 7 program written specifically for these simulations. This program can run arbitrarily configured stochastic reaction-diffusion systems (S1 Program ZIP archive file includes the application program, source code, examples, and documentation). The program provides a graphical user interface developed using NetBeans 7.4 IDE and Java Swing (Oracle) to define the reaction-diffusion system, initialize it, and run the SSA. Capabilities are in place to save data and images of the system states, and QuickTime-formatted movies of the simulation can be produced. The model simulation state can be saved, so the simulation can be stopped and continued at any point in time. As a Java program, it can be run on any platform that supports Java 7 or later.

#### Model with 3D structure

**Fig 3:**
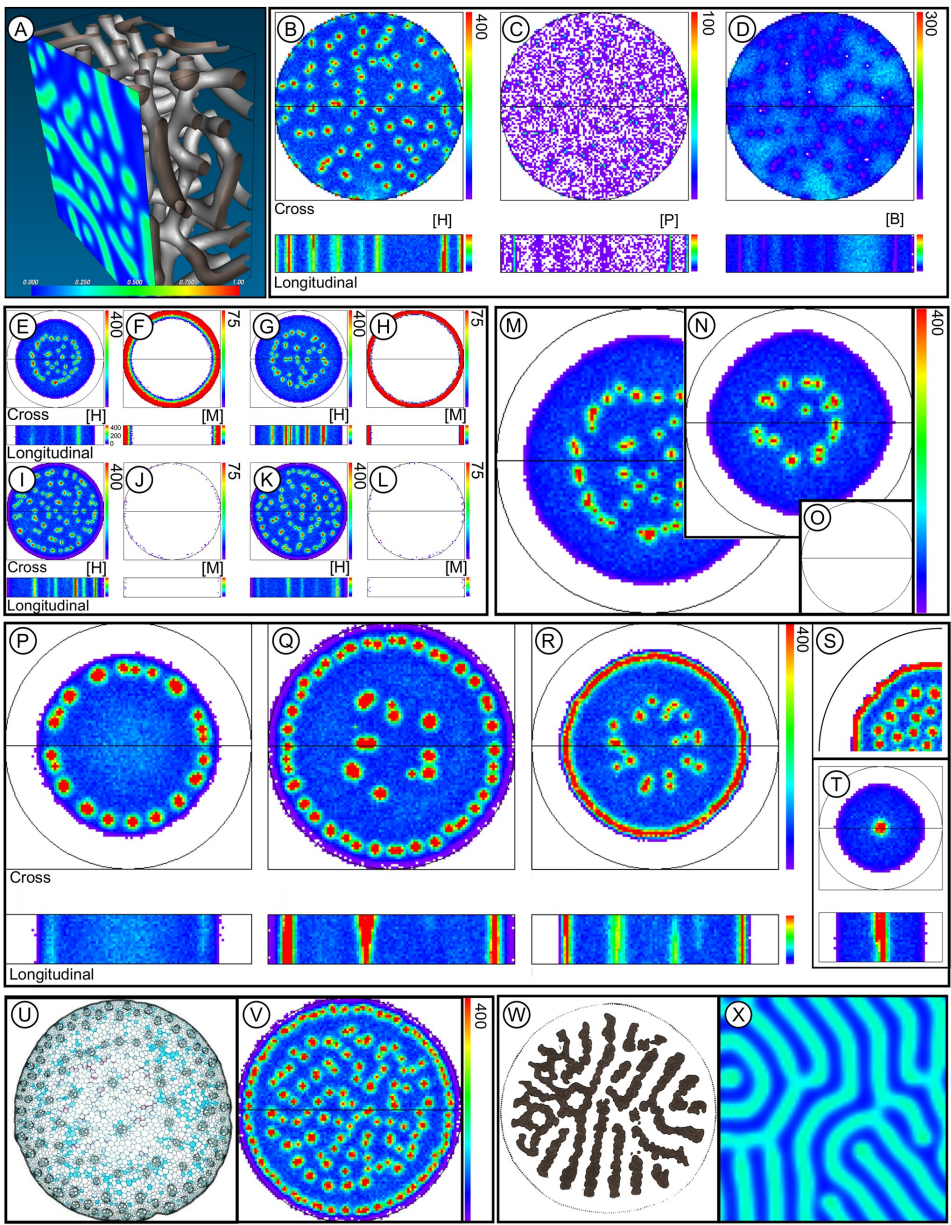
Model development and simulation results. This figure illustrates results of simulations from the two-molecule *HB* model through to the four-molecule *HBPM* model. The text guides through the incremental addition of molecules to the model. S1 and S2 Tables and S1 through S19 Movies provide simulation parameters and visualizations, respectively. (A) The two-molecule (*HB*) model in 3D. Under appropriate parameterizations (S1 Model provides parameter values and simulates the *HB* model when run by the program Ready v. 0.8), the Gray-Scott-Schnakenberg model produces a network of vessels with high [*H*], but longitudinally-oriented vessels are largely absent. The color image represents a cross-section with color intensities scaled between least (0.000) and maximal (1.00) concentrations. (B)-(V) Black circles represent edges of the stems, and black lines across circular cross sections show where longitudinal sections were made in 3D models. Color bars show concentrations (in numbers of molecules per voxel) with the maximum concentration value shown ranging down to 0 at the bottom of the color bar. White color is background color, representing the absence of molecules. (B)-(D) The *HBP* model with a longitudinal flux transporter, *P*, results in spots of high [*H*] in cross section and longitudinally-oriented cylinders of high [*H*] in longitudinal section, akin to plant stem vessels. (E)-(L) *HBPM* model showing suppressive effects of *M* on the spatial distribution of supplemental vascular bundles. Production of *M* at the periphery of the stem restricts the SVBs to the central region of the stems. (E)-(F) High *M* production, high rate of *M* diffusion. (G)-(H) High *M* production, low rate of *M* diffusion. (I)-(J) Low *M* production, high rate of *M* diffusion. (K)-(L) Low *M* production, low rate of *M* diffusion. Panels (M)-(T) display [*H*]. (M)-(O) Gain and loss of SVBs with changes in stem size. (M) Large diameter stem. (N) Medium diameter stem is 75% of the diameter of the large diameter. (O) Small diameter stem is 37% of the diameter of the large stem. *M* suppresses vascular bundle formation in the narrowest stem. (P)-(V) Simulation results that resemble stelar patterning in plants. The *HBPM* model was used and simulated [*H*] is shown in the figures. (P) Eustele pattern. (Q) Eustele pattern with SVB patterns internally. (R)-(S) Siphonostele pattern with SVB patterns internally. (T) Haplostele pattern. (U)-(V) Monocot stem patterning. Atactostele vessel patterning similar to those of monocots, such as *Zea mays*, in (U), illustrating a uniform ring of bundles at the stem periphery and scattered bundles in the stem center. (W)-(X) Lycopod stem patterning. (W) *Lycopodium scariosum* stem in cross section illustrating a plectostele. (X) 2D deterministic simulation of the Gray-Scott model recreating the plectostele pattern. S2 Model file provides a plectostele model file which runs in the program Ready v. 0.5. Panel (W) adapted from [46].

The two-molecule *HB* model produced spots of high [*H*] that were regularly arrayed within the simulated 2D stem cross section, as expected (see, e.g., [37]). In these simulations, these regions of high [*H*] represent regions of vascular tissue differentiation. Running the deterministic Gray-Scott-Schnakenberg reactions in 3D in the software package Ready v. 0.8 [45] resulted in a connected network of conduits with high [*H*] (Fig 3A; S1 Table, Simulation A; S1 Model), whereas stochastic implementations of Eqs 1 and 2 resulted in disconnected regions of high [*H*] in 3D (S1 Movie provides one realization of the 3D implementation of the *HB* model). Neither the stochastic nor the deterministic approaches reproduced the longitudinal orientation of vascular bundles seen in plant stems.

To improve the model of longitudinal development of vascular bundles, the *HB* model was augmented to include an additional molecule–an efflux carrier molecule, labeled *P*. The *HBP* model is represented by Reactions *X*_*1*_ –*X*_*8*_ of Table 1 in which *P* transported *H* longitudinally (reaction *X*_*8*_: *P*↑↓*H*). *H*, in turn, promoted expression of *P* (reaction *X*_*6*_: *H*→*H*+*P*). All parameterizations of the 3D simulations presented here that included *P* are in S1 Table and S2 Table.

Molecular transport via carrier molecule *P* can be shown to follow Michaelis-Menten kinetics as follows. During passive diffusion, a molecule that diffuses outside of its voxel is equally likely to diffuse into any of the 6 adjacent voxels. Two of these six are in the longitudinal orientation, so a molecule that is passively diffusing out of its voxel will diffuse longitudinally with a probability of 1/3. When *P* is included in the model, the probability that a molecule of *H* that is leaving its voxel, *i*, moves longitudinally (↑↓*H*) was modeled as (Eq 3):

$${Probability(↑↓H)}_{i}=\frac{Propensity\;for\;longitudinal\;movement\;from\;voxel\;i}{Total\;movement\;propensity\;from\;voxel\;i}=\frac{2D_{H}+\alpha {\left[P\right]}_{i}}{6D_{H}+\alpha {\left[P\right]}_{i}}=\frac{\frac{2}{3}{\left[P\right]}_{i}}{\frac{6D_{H}}{\alpha }+{\left[P\right]}_{i}}+1/3$$ (Eq 3)

Here, *α* can be thought of as *H*’s affinity for *P* and *D*_*H*_ is the coefficient of passive diffusion for *H*. Setting the Michaelis-Menten parameter *V*_*max*_ to 2/3 and the Michaelis-Menten parameter *K*_*m*_ to 6*D*_*H*_/*α*, the probability that *H* moves longitudinally is seen to obey Michaelis-Menten kinetics above the background passive longitudinal diffusion probability of 1/3 by comparison to the Michaelis-Menten equation.

#### Refining spatial pattern through inhibition of *H* by *M*

**Fig 4:**
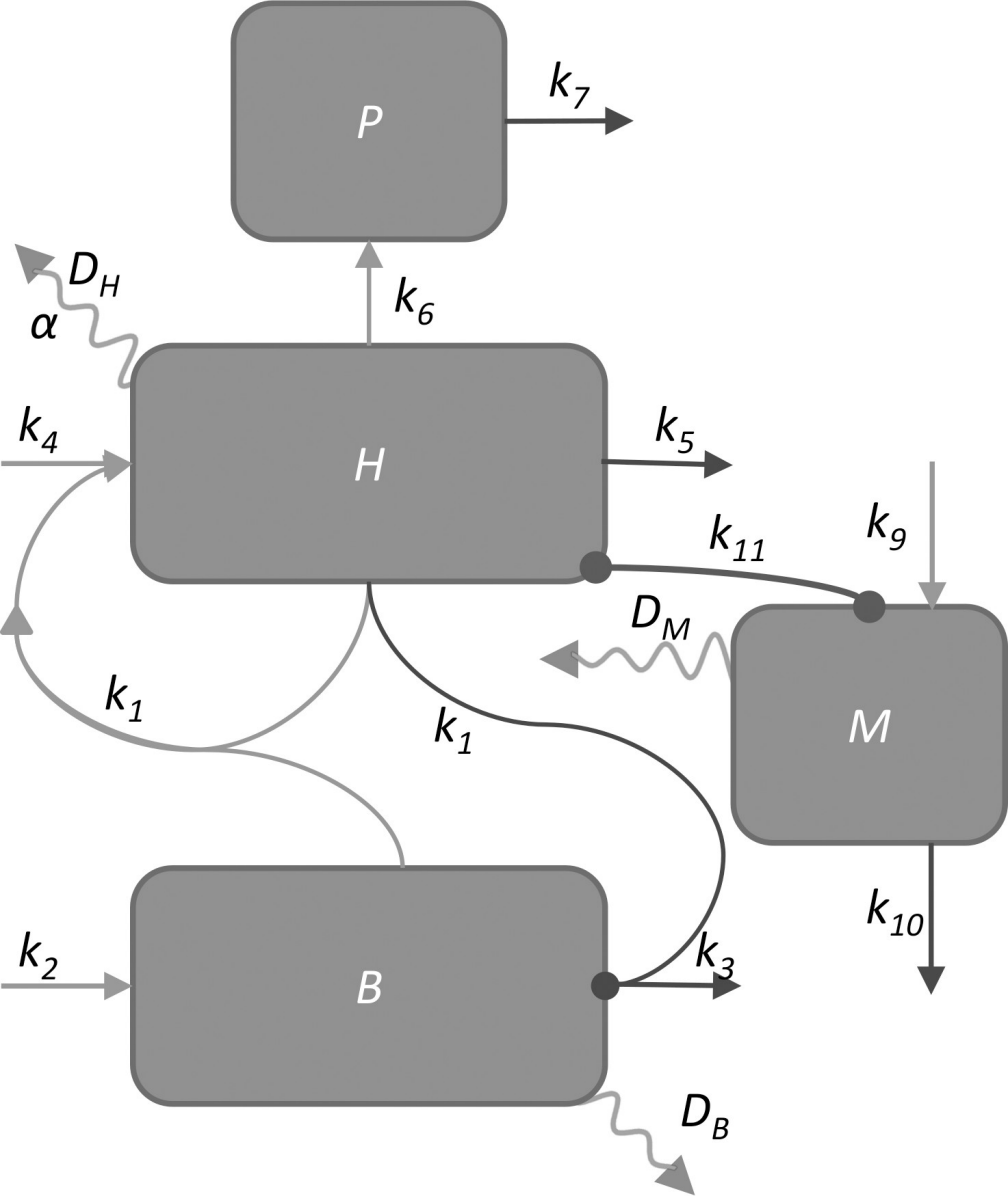
*HBPM* model diagram. Parameter symbols are as in the Table 1. Dark lines with circle end points indicate suppression in the direction of the circle. Arrowed lines indicate activation in the direction of the arrow. Arrows with only one connected end represent constitutive or background production (arrow pointing to box) or degradation (arrow pointing away from box). Squiggly arrows represent diffusion. Some simulations also included mutual inhibition between *M* and *B*, but this was removed in later simulations.

The augmented model in which *P* polarized *H* longitudinally produced longitudinally oriented bundles (Fig 3B–3D; S2 Movie) throughout the stem. In eudicot stems, however, medullary SVBs tend to occur in the centers of stems with a zone of separation between them and the secondary vascular cambium (Fig 2 region B). The three-molecule model (*HBP*) was further augmented to better match observed distributions of SVBs by including an inhibitor molecule, *M*. This model that includes four molecules *H*, *B*, *P*, and *M* (hereafter called the *HBPM* model; Fig 4) is represented by Reactions *X*_*1*_ –*X*_*12*_ of Table 1. *M* was produced in the outer 4% of the simulated stem and diffused passively throughout the stem. Its interaction with *H* resulted in the degradation of both *H* and *M* (Reaction *X*_*11*_: *M*+*H*→Ø).

#### Additional elaborations of the *HBPM* model

To further increase biological realism, a final iteration of the *HBPM* model added an additional molecule, *A*, which was produced exclusively at the top layer of the simulated stem (apex) and interacted with *H* in a regulatory feedback loop. In the augmented model, *A*, rather than *H*, was transported by *P* basipetally and upward facilitated transport did not occur. This augmented model did not involve any additional pattern formation mechanisms compared to the *HBPM* model, but it incorporated source-sink dynamics in plant vascular pattern formation in which the stem apex was the source of *A*. This augmented *HBPM* model is detailed in S2 Table.

### Examining hypotheses in silico with the *HBPM* model

#### Effect of stem diameter on formation of vascular bundles

SVBs are most frequently found in wide parenchymatous stems, hypocotyls, or roots, and they tend to be absent from thin ones [29,30,47]. Can reductions in stem diameter–and in particular changes in size of the shoot apex near to where vascular patterning is initiated by procambia—explain the absence of SVBs in narrow stems and their development in wider stems? In narrow stems, *M* was predicted to diffuse into stem centers and suppress *H* (and therefore SVB) production throughout the stem. In contrast, *H* was predicted to be active in the centers of thick structures where *M* does not reach, since *M* is depleted (reactions *X*_*10*_ and *X*_*11*_, Table 1) while it diffuses from the cortical site of production toward the stem center (Fig 2).

To investigate the effect of rates of diffusion and production of *M* on the localization of SVBs, two rates of diffusion and two rates of production were explored (S1 Table, Simulations 3–6, parameters *D*_*M*_ and *k*_*9*_). Simulations 3 and 7–9 (S1 Table) explored the impact of varying the stem radius while keeping all other rate and diffusion parameters constant.

#### Effect of diffusion rates of *H* and *B* on density and size of vascular bundles

To investigate how diffusion rates of *H* and *B* may influence size and density of SVBs, their diffusion rates were varied while other reaction rate parameters were kept constant. Following the results of Wei and Winter [37], it was expected that higher diffusion rates of *D*_*H*_ would result in wider simulated vascular bundles and higher *D*_*B*_/*D*_*H*_ ratios would tend to produce lower densities of simulated vascular bundles. For these simulations, the 2D *HB* model that did not include *M* and *P* was used (i.e., Eqs 1 and 2) in order to focus on the role that diffusion rates of *H* and *B* play in patterning. *D*_*B*_/*D*_*H*_ ratios were set to 10, 100, 1000, 2000, 2500, 3000, 3500, and 4000, and *D*_*H*_ was set to 0.016 or 0.05. The SSA was run three times for each combination of parameters, and the regions of high [*H*] were counted, measured, and graphed as a function of *D*_*B*_/*D*_*H*_ vs. *D*_*H*_ once stationarity was reached.

Simulations typically took several days to reach stationarity. 2D models run more quickly than 3D models and therefore permit a more rapid exploration of model parameter space. However, to check that the expectations concerning diffusion rate and size and density of simulated SVBs were met in a 3D model, three 3D simulations (Simulations 12–14, S1 Table) of the *HBPM* model were run with *D*_*H*_ set to 0.02, 0.0375, and 0.075 and *D*_*B*_/*D*_*H*_ ratios correspondingly set to 100, 66.67 and 375.

### *HBPM* modeling of stelar patterning

During the course of developing the *HBPM* model for the above simulations, multiple combinations of parameters were explored with the goal of determining the range of applicability of the model to the general patterning of vascular steles. Parameters used during these simulations (Simulations 15–18) are described in detail in S1 Table.

## Results

### Model creation

In deterministic versions of the Gray-Scott-Schnakenberg model that produce stationary regions of high [*H*], these regions tend to be uniformly spaced. In the 3D *HBPM* model, *P* appeared to canalize and spatially fix the more randomly dispersed conduits of high [*H*]. Simulation 1 was run for 279,104,164,995 reaction events after which the pattern of [*H*] did not change substantially (Fig 3B–3D). In 2D simulations that lacked *P*, this canalization of high [*H*] did not occur, and regions of high [*H*] moved until uniformly spaced (e.g., S11 Movie).

The original *HBPM* model was augmented to include *A*, representing auxin, which is apically produced and transported basipetally in the model. Results of two simulations of the augmented model are presented. S19 Movie presents the outcome of a simulation with apical production of *A*, basipital transport of *A* via *P*, and diffusion of *H*, whereas S1 Fig presents the outcome of the same augmented model, but with no diffusion of *H*.

### Examining hypotheses in silico with the *HBPM* model

#### Effect of stem diameter on formation of vascular bundles

Simulations 3–6 explored the impact of *M* by varying its rates of production and diffusion within a constant diameter stem. These simulations all produced longitudinally oriented SVBs and zones that lacked SVBs that corresponded to zone B of Fig 2. The rate of production of *M* most highly impacted the width of zone B (Fig 3E–3L; S3–S6 Movies).

To examine the impact of changes in stem diameter on SVB patterning, reaction parameters were held constant in narrow, medium, and wide stems. Simulation of a wide diameter stem resulted in scattered SVBs that were restricted to the central part of the stem. There were a few, scattered SVBs in medium-diameter stems, and *M* suppressed the production of SVBs in narrow stems (Fig 3M–3O; S3 and S7–S9 Movies).

#### Effect of diffusion rates of *H* and *B* on density and size of vascular bundles

**Fig 5:**
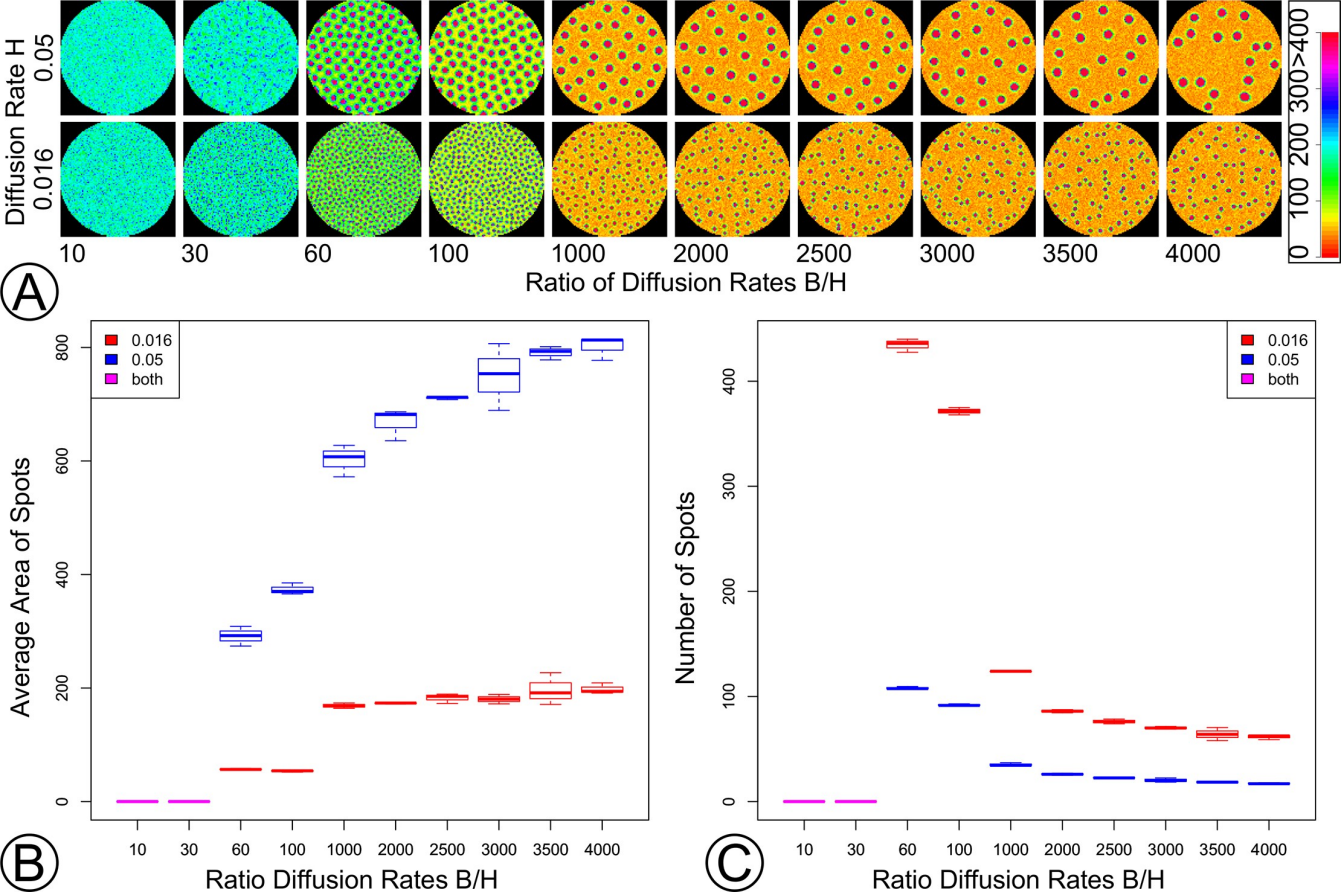
Density and size of supplemental vascular bundles varies with diffusion rates of *H* and *B*. (A) [*H*] for various diffusion rates of *H* (*y*-axis) and ratio of diffusion rates of *B* and *H* (*D*_*B*_/*D*_*H*_) (*x*-axis). Legend shows mapping between [*H*] and color (Black represents no molecules). (B) Diameter of regions of high [*H*] (>400 molecules per voxel) as a function of *D*_*B*_/*D*_*H*_. (C) Number of regions of high [*H*] as a function of *D*_*B*_/*D*_*H*_. Legends for (B) and (C) show color coding for diffusion rates of *H*. Note that *x*-axes are not to scale in (B) and (C). Box plots are based on 3 simulation replicates of each parameter combination. S10 and S11 Movies provide example animations of *H* diffusion rate at 0.05 and ratios at 10 and 1000, respectively.

Holding all other parameters constant while varying the diffusion rates of *H* and *B* resulted in changes to the number and widths of simulated vascular bundles. As predicted, changes to the diffusion rate of *H* (*y*-axis, Fig 5A) in large part accounted for variation in spot size, whereas variation in the ratio *D*_*B*_/*D*_*H*_ (*x*-axis, Fig 5A) in large part accounted for changes in spot density (Fig 5; S10 and S11 Movies provide example 2D simulations with contrasting *H* and *B* diffusion parameter combinations). However, density and spot size were not independent, and spot sizes tended to decrease with increased spot density, even for constant diffusion rate of *H* and varying diffusion rate of *B*. Low diffusion rates of *H* resulted in spatially noisy [*H*] and no persistent regions of high [*H*] (S10 Movie).

All simulations examining diffusion rates of *H* and *B* were carried out in 2D did not include *M* and *P*. Without *P*, the longitudinal canalization effect was not present and spots spread uniformly throughout cross sections in the absence of *M* (S11 Movie). 3D simulations of the *HDPM* model with varying *D*_*H*_ and *D*_*B*_ matched expectations. Higher diffusion rates of *H* resulted in larger zones of high [*H*], and smaller spots tended to be in higher density than larger spots, even for larger *D*_*B*_/*D*_*H*_ ratios. (S12–S14 Movies, S1 Table).

### *HBPM* modeling of stelar patterning

The *HBPM* model recreated multiple stelar patterns with or without SVBs. A ring of vascular bundles (eustele) typical of the primary state of growth in eudicots resulted when *B* was degraded at a low background rate (Fig 3P; S15 Movie). When the degradation rate was further lowered and production of *M* decreased, a ring of radially-arranged vascular bundles with central SVBs formed (Fig 3Q; S16 Movie); the patterning of vascular bundles resembled the A (cortex), B (lack of SVBs) and C (presence of SVBs in pith) zonation of kohlrabi (Fig 2B). When background degradation of *B* was removed, a vascular cylinder (siphonostele) was produced with SVBs internally (Fig 3R; S17 Movie). The *HBPM* model established both the positioning of the secondary vascular cambium at the stem periphery at the interface between zones A and B, and it recreated the centrally-located SVBs. The zone B that lacks SVBs appeared to result from depletion of *M* and *B* in region B caused by the formation of the radially oriented bundles at the stem periphery. As a result, the core reaction that enhanced production of *H* (*X*_*1*_ of Table 1) could not occur in zone B where [*B*] was depleted.

Additional stelar patterns were observed under different parameterizations. In the simulation of a narrow stem, the results resembled a central rod of vascular tissue (haplostele) (Fig 3T; S8 Movie). Such a protostele is typical of roots with narrow root apices and represents another instance of how changes to boundary conditions influence vascular patterning. When suppression by *M* was reduced, a layout qualitatively similar to that of some monocots (atactostele) resulted in a uniform ring of high [*H*] that resembles vascular bundles peripherally, and scattered bundles internally (Fig 3V; S18 Movie). Finally, a deterministic version of the Gray-Scott model, Simulation B, produced patterns in simulated [*H*] that resembled plectosteles in stems of some lycopods (Fig 3W and 3X; S2 Model file for use with Ready 0.8 program).

## Discussion

The *HBPM* model recreated three dimensional plant stem vascular patterning using a Turing-like mechanism in a stochastic, reaction-diffusion framework. Vascular patterns it recreated included atactostele, plectostele, haplostele, siphonostele (continuous vascular cylinder), and eustele (siphonostele with vascular bundles arranged radially) arrangements (Fig 3). Additionally, the model recreated patterning of a cambial variant in which supplemental vascular bundles (SVBs) develop in the center of the stem. As a mathematical model, it requires further grounding in molecular biological details, and it offers several testable predictions about evolutionary-developmental mechanisms of pattern formation.

### Evolutionary-developmental hypotheses stemming from simulated outcomes of the *HBPM* model

Multiple sets of simulations explored how (1) changes in stem size (boundary conditions), (2) changes to the spatial expression of an inhibitory molecule (*M*), and (3) changes to diffusion rates of *H* (the molecule triggering vascular development) and *B* (its substrate) can influence vascular patterning.

(1) Changes to stem diameter altered SVB patterning in the *HBPM* model. Wide stems tended to develop longitudinally-oriented SVBs in their centers whereas narrow stems lacked SVBs. In these simulations, no changes to reaction, production, degradation, and diffusion rates were made; only stem diameters were altered. Empirically, Torrey [48,49] found that stelar patterns in pea roots are largely a function of the diameters of apical regions.

One implication of the simulations that modeled changes to stem diameter is that evolutionary gains or losses of a trait may have little to do with evolutionary changes to gene expression rates or functions of the genes responsible for the development of a trait. Instead, losses, for example, may be due to evolutionary changes in boundary conditions that allow a diffusible inhibitor to reach the location of pattern formation that was previously inaccessible to the inhibitor. This scenario can explain the loss or gain of SVBs in the *HBPM* model when stem diameter is varied. When (simulated) evolution decreases stem girth (through other developmental pathways), SVBs fail to develop due to inhibition of *H* by *M*, whereas (simulated) evolution of wide-stemmed plants in which the inhibitor molecule does not reach the centers of stems will result in SVB development (compare Fig 3M–3O). So, changes to genes responsible for the evolutionary loss or gain of a trait may be independent from the molecular-developmental pathways responsible for the trait’s development.

(2) Changes in spatial expression of the inhibitor, *M*, altered the position of the stele and vascular bundles in relation to the edge of the stem (Fig 3E–3L). With no expression of *M*, the atactostele pattern found in wide-stemmed monocots was the result. Evolution can cause such changes by altering promotor regions, thereby influencing the rate and spatial positioning of expression. Carroll and others [1,50] argue that many changes to organismal patterning are due to such mechanisms.

(3) Changes in diffusion rate of *H* and *B* altered the number and size of SVBs even when all other aspects of the model (e.g., reaction kinetics, production and degradation rates, etc.) remained constant (Fig 5). Moreover, simulations in which diffusion rates of *H* fell below a threshold rate failed to develop vascular bundles altogether (S10 Movie). Mathematical theory suggests that inhibitor molecules must diffuse at least six to seven times faster than activator molecules in order for heterogeneous patterns of the activator molecule to emerge ([51] and p. 246 in [52]).

A broader hypothesis that follows from this modeled result is that phenotypic diversification may occur as a consequence of evolutionary changes to protein movement rates. The logic of this hypothesis is as follows: (1) Evolution can influence lengths of proteins through insertion or deletion (indel) mutations; (2) changes in protein lengths influence their rates of movement (smaller molecules tend to move faster); (3) changes in rates of molecular movement can impact patterning processes during development. Therefore, evolution may be able to alter patterning by changing molecular movement rates. The first statement follows by definition of indel mutations. The second statement follows from basic thermodynamics and is empirically supported by some relatively recent work that tracked individual, tagged molecules. This research found that protein size is the primary determinant of cytosolic diffusion rate in *E*. *coli* cells [53]; Nenninger et al. [53] also found that, empirically, the coefficient of diffusion is related to the size of the protein under constant temperature and fluid viscosity via the Stokes-Einstein formula. Finally, the third statement has been recognized since Turing’s 1952 paper [25]. If molecular movement rates are largely a function of molecular size, then even in the absence of selection for change in amino acid composition and protein function, variation in protein size can play a role in phenotypic evolution through changes in movement dynamics. This relationship between protein size and patterning of serially homologous features also raises the intriguing possibility that different alternative splice isoforms of a gene’s transcript permit fine tuning of diffusion rates–and hence pattern formation processes–in specific cell lineages.

Interestingly, Lipman et al. [54] discovered that length variation is highest among the most conserved, “more important” (their words) proteins. They interpreted protein size variation in terms of transcriptional costs, and they also suggested that size variation in conserved proteins reflects divergence in protein function. Selection on protein diffusion rate provides an alternative interpretation in which the fine tuning of protein length (hence diffusion rate) and not protein function, *per se*, influences the outcomes of spatio-temporal developmental patterning and selection on phenotypes.

### Biological assumptions and possible molecular bases of the *HBPM* model

In order to design empirical experiments to test the above hypotheses based on the *HBPM* model, the components of the model must be empirically grounded with molecules of relevance to vascular patterning in plants. Mathematical modeling and molecular-genetic research about plant vascular development have long suggested that members of the *Class III HOMEODOMAIN-LEUCINE ZIPPER* (*HD-ZIP III*) gene family are essential for the initial specification of growth regions (procambia) that develop into xylem and phloem [5,55–61] through regulatory feedback with auxin [62], KANADI [60], brassinosteroid (BR) [4,63], and possibly with Class I KNOTTED-LIKE HOMEOBOX (KNOX) transcription factors [64].

The model of Benítez and Hejátko [5] required positive autoregulation of an HD-ZIP III gene (*HB8* in Benítez and Hejátko) in coordination with another molecule for vascular patterning to emerge. A similar model of autocatalysis has been proposed for at least one other *HD-ZIP III* gene family member, *PHABULOSA* (*PHB*), in which a ligand for PHB activates the PHB protein and may increase its expression [65]. Empirically, the *HD-ZIP III* gene *HB8* is upregulated in regions of tuberous stems and roots with SVBs in *Brassica* crops [36]. In the *HBPM* model, *H*, appears to play the role of an HD-ZIP III protein involved in procambial specification and xylem differentiation, whereas *B* plays the role of the ligand involved in *H*’s autoregulation (Table 1 Reaction *X*_*1*_). HD-ZIP III transcription factors are active as homodimers [66] consistent with *H*’s dimeric interaction in the core *HB* model (Eq’s 1 and 2). *B* remains an unspecified positive regulator of the *HD-ZIP III* protein that is itself degraded by the *HD-ZIP III* protein. This regulator of the *HD-ZIP III* gene may be BR, an ARF such as MP, auxin, a KNOX I transcription factor, or an unidentified molecule [67]. Ibañes et al. [4] suggested that BR is essential for vascular bundle formation in *Arabidopsis*, and Benítez and Hejátko [5] postulated that *HB8* self-upregulates via BR. In the current model, *B* may play the role of BR or a regulator of BR such as a member of the *BRL* gene family [63].

Ibañes et al. [4] also suggested that polar auxin transport is required for the patterning of vascular bundles. Sachs [68–70] was the first to suggest that the self-reinforcement of auxin through polar transport canalizes auxin at the site of procambial strand initiation. Basal positioning of the auxin efflux protein *PIN1* within cells via gravity-sensing mechanisms [71–73] precedes this canalization effect [21,60,74,75]. Specification of procambial cells occurs through the activities of auxin response factors (*ARF*s) such as MP and the induction of HD-ZIP III *HB8* expression [62] in a feedback loop. Moreover, in the model of Muraro et al. [7], an HD-ZIP III gene (*PHABULOSA*) was an indirect positive regulator of *PIN* proteins, and *ARF*s were indirect positive regulators of *HB8* in Benítez and Hejátko’s [5] model. Furthermore, *HD-ZIP III* genes can promote expression of the PIN-FORMED (PIN) efflux carrier proteins [62,74] that contribute to polar auxin transport in a complex network involving positive regulation of *HD-ZIP III* transcription factor genes (e.g., *HB8*) by the auxin response transcription factor (ARF), MONOPTEROS (MP) (reviewed by, e.g., [12]). During simulations of the *HBPM* model, no parameter combinations were found that recreated the patterning of longitudinally-oriented vascular bundles in 3D without simulating an efflux molecule, *P*. Based on the above considerations, *P* matches the function of PIN1.

Lastly, how does *M* function in the *HBPM* model? Empirically, microRNA 165/166 depletes HD-ZIP III and thereby limits the development of vascular tissue [76]. *M* performs the function of microRNA 165/166 in the *HBPM* model. In roots, SHORT ROOT (SHR) and SCARECROW (SCR) that accumulate in the endodermis increase microRNA 165/166 production in the endodermis (reviewed by [77]). In the *HBPM* model, expression of *M* is restricted to the stem cortex *a priori*, analogous to how its expression is restricted to the endodermis in roots [7]. However, the empirical spatial distribution of microRNA 165/166 in stems is less well understood. The model suggests that miRNA165/166 suppression of an HD-ZIP III protein may restrict the differentiation of SVBs to the central regions of plant structures and foster the partitioning of stems into the familiar cortex-cambium-xylem-pith configuration.

### Addressing biological assumptions

If, indeed, *H*, *B*, *P*, and *M* correspond to an HD-ZIP III protein, a brassinosteroid (or unknown molecule), a PIN efflux protein, and microRNA 165/166, respectively, then the initial *HBPM* model needs refinement to better correspond to biological reality. First, PIN1 transports auxin and is very unlikely to transport HD-ZIP III proteins. Second, auxin is known to be produced apically and it is transported primarily basipetally. Third, HD-ZIP III proteins are quite large (~91kDA), and it is unlikely that they can diffuse freely between cells, even through plasmodesmata [78]. To increase biological realism, *HBPM* was augmented to model auxin, symbolized as *A*, explicitly. The model was reconfigured so that *A*, and not *H*, was transported basipetally by *P*. The simulated expression of *A* was also restricted to the top layer of the simulated stem to model apical expression of auxin. *H* and *A* were simulated to regulate each other in a feedback loop in the augmented model. These additional reactions are presented in S2 Table.

In the augmented model simulations, the concentration of [*H*] remained high in the apical region. Below this region of high concentration was a region of low concentration, and under this region, longitudinally oriented regions of high [*H*] occurred matching the layout of procambial patterning in some plants (the discontinuity in S19 Movie corresponds to a shift of view from the top layer of high concentration to the lower layer in which longitudinal patterning of high [*H*] developed).

When the diffusion rate of *H* was set to 0 to limit its diffusion due to its large size, longitudinally arranged regions of high [*H*] resulted, but these were irregularly spaced in the stem and had narrow diameters. In all augmented simulations with *A*, longitudinally arranged regions of high *H* were crooked, unlike those in the “un-augmented” *HBPM* simulations.

So, the *HBPM* model captures a lot of the variation and patterning in plant stem vascular tissue, but it remains incomplete. The actual regulatory network underlying vascular development is substantially more complicated than a four- or five-molecule model, and additional components are required to further refine the model of three-dimensional vascular patterning.

In particular, longitudinal, cellular growth near the apex and in rib meristems was ignored. Gene expression states of cells could be epigenetically inherited during cell division in these regions resulting in longitudinally oriented vessels without PIN proteins. Future modelling efforts should include growth dynamics to examine how apical growth may contribute to three dimensional patterning of vascular tissue in stems.

### *HBPM* model predictions

Further functional molecular genetic validation of the *HBPM* model is required. This model makes multiple predictions that can be tested in the wet-lab:

1. Production of miRNA165/166 is highest at the stem periphery, and production of miRNA165/166 may be reduced in monocot stems with atactosteles.
2. Release of *HB8* or other *HD-ZIP III* genes from suppression by miRNA165/166 will increase the zone of SVB production throughout the stem in stems composed of ground tissue or parenchyma, more generally (c.f. [58]).
3. Suppression of *HB8* expression (or possibly a different *HD-ZIP III* paralog) will annihilate SVB development.

Different parameterizations of the *HBPM* model led to patterning of vascular bundles that resembled monocot vascular bundle patterning (Fig 3V) or eudicot patterning (Fig 3P) and recreated the partitioning of stem into cortex, vascular cambium, and pith. *M* influenced the relative sizes of these regions, so altering the location and expression rate of microRNA 165/166 is also predicted to influence the relative sizes of cortex, vascular regions, and pith in the stem.

## Conclusions

The *HBMP* model and its augmentations demonstrate that, in principal, an activator-substrate-inhibitor Turing-like patterning mechanism with longitudinal transport via Michaelis-Menten kinetics of a regulatory molecule is sufficient to canalize the differentiation of radially-patterned, longitudinally-oriented vascular bundles within stem ground tissues. The result that a single reaction network can recreate most of the common vascular patterns in plant stems raises the possibility that a single conserved genetic network regulates stem vascular patterning in plants more broadly and that evolution may “configure” this network within particular lineages by altering boundary conditions and reaction-diffusion parameters.

Although incomplete, the augmented *HBMPA* model appears to correspond best with activities of HD-ZIP III regulatory gene products (*H*), miRNA inhibitors of *H* (*M*), PIN1 efflux carrier proteins (*P*), auxin (*A*), and an unknown substrate (or cofactor) of *H* (hypothesized to be a brassinosteroid, *B*). The HD-ZIP III genes comprise an ancient family of genes that are active in non-vascular and vascular land plants, and they appear to be involved in vascular development broadly throughout the vascular plants. In animals, it is increasingly becoming apparent that evolutionary tweaking of the temporal and spatial expression of conserved “toolkit” genes [50] underlies animal diversity. Is there a “toolkit” for vascular development in plants? If so, HD-ZIP III gene products and molecules interacting with them are likely to be important components of the core “kit”.

**Fig 6:**
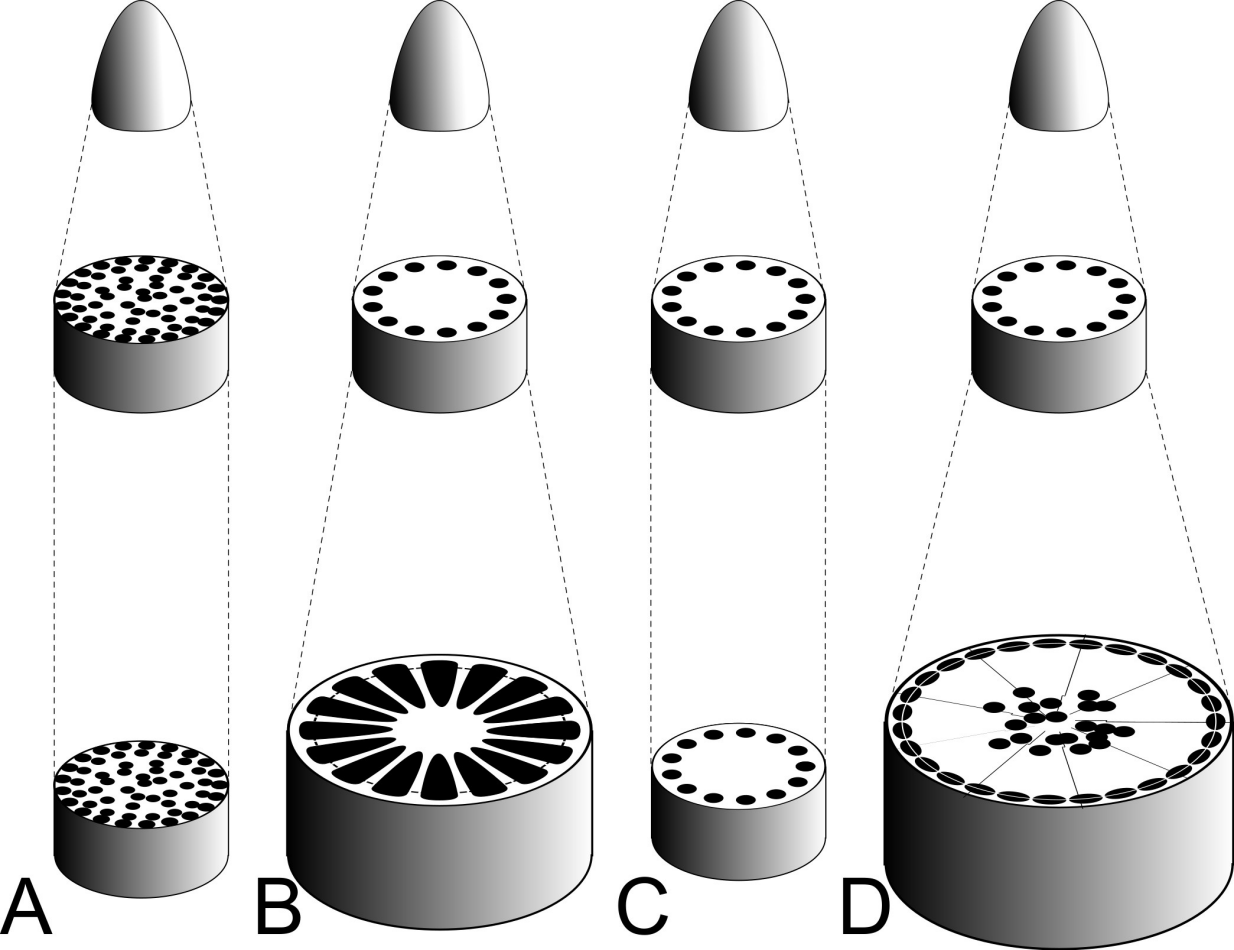
Evolutionary-developmental model of stem vasculature. When seen in the context of the entire plant stem, the *HBPM* model (Fig 4, Table 1) accounts for much variation in plant stem vascular structure. In each panel of the figure, the top represents the shoot apex, followed by a cross section with the primary state of growth (compare to Fig 3P), followed by a cross section of the mature stem. (A) Monocot stem vascular patterning as seen in corn or bamboo (compare to Fig 3V). (B) Woody plant with primary vascular bundles that merge and develop into the vascular cambium which produces lignified xylem (black sectors). Reduced axial parenchyma and pith may impose limits the production of SVBs although these are occasionally found in pith of woody plants [27]. (C) An herbaceous eudicot maintains the primary state of growth with limited cambial development and limited increase in stem girth. Narrow stems restrict the production of SVBs (compare to Fig 3O and 3P). (D) A stem-succulent plant with extensive parenchymatous wood. The primary vasculature transitions into a secondary vascular cambium that produces parenchyma primarily. *H* and *B* interact in wide zones of parenchyma to produce SVBs in the stem centers away from the suppressive effects of *M* (compare to Fig 2B and Fig 4Q–4S).

The current model provides a unified framework that suggests that shared, homologous, deeply conserved molecular genetic machinery is sufficient to explain diverse stem vascular patterns (Fig 6). However, the molecular genetic details of the *HBPM* model and its evolutionary-developmental implications concerning the fine-tuning of biological patterning via evolution of diffusion rates and reaction-diffusion boundary conditions require further empirical validation and scrutiny.

## Supporting information

S1 Appendix Algorithmic details of the stochastic reaction-diffusion simulation. (DOCX)

S1 Model Simulation A parameter file for the program Ready 0.8 [45] to simulate Fig 3A. (VTI)

S2 Model Simulation B parameter file for the program Ready 0.8 [45] to simulate Fig 3X. (VTI)

S1 Program Executable Java archive file (.jar) to run simulations. This file is a ZIP archive file of multiple files. In addition to the executable jar file, the ZIP file includes a tutorial, source code in the .jar file, and java library files (lib directory) required to run the reaction-diffusion program.
(ZIP)

S1 Table Simulation parameters. Column headers indicate the simulation lattice dimensions, the reactions (from Table 1) and their rate parameters, and the diffusion parameters. S1–S18 Movies file names and figure numbers are also provided to see visualizations of the simulations. The columns “radius *M*” and “width *M*” describe the radius and thickness of the cylindrical shell in which *M* is constitutively expressed.
(XLSX)

S2 Table Augmented *HBPM* model with apical production of *A* and model parameters. This table includes parameters for S19 Movie and associated simulation.
(XLSX)

S1 Movie Simulation 1 Three dimensional simulation of the HB model. Movie files are QuickTime-formatted movie files that visualize the time course of simulation data. Parameter values for corresponding simulations are in S1 and S2 Tables. The figure number that visualizes the end result of the simulation is provided next to each movie file name. In all movies, the top, middle, and bottom row of each column represent the concentrations of reactants in cross-section, longitudinal section, and total concentration over time, respectively. Color bars follow conventions described in Fig 3.
(MOV)

S2 Movie Simulation 2 lacking M. Fig 3B–3D. See S1 Movie legend for additional details.
(MOV)

S3 Movie Simulation 3 with high M production rate and high M diffusion rate. Fig 3E and 3F. See S1 Movie legend for additional details.
(MOV)

S4 Movie Simulation 4 with high M production rate and low M diffusion rate. Fig 3G and 3H. See S1 Movie legend for additional details.
(MOV)

S5 Movie Simulation 5 with low M production rate and high M diffusion rate. Fig 3I and 3J. See S1 Movie legend for additional details.
(MOV)

S6 Movie Simulation 6 with low M production rate and low M diffusion rate. Fig 3K and 3L. See S1 Movie legend for additional details.
(MOV)

S7 Movie Simulation 7 with 75% stem diameter. Fig 3N. Simulation 2 (S2 Movie) provides the reference stem with 100% diameter. See S1 Movie legend for additional details.
(MOV)

S8 Movie Simulation 8 with 50% stem diameter and patterning resembling a haplostele. Fig 3T. See S1 Movie legend for additional details.
(MOV)

S9 Movie Simulation 9 with 37% stem diameter. Fig 3O. See S1 Movie legend for additional details.
(MOV)

S10 Movie Simulation 10 in 2D of the two-molecule HB model. This simulation generates the Fig 5A panel with the lowest *D*_*B*_/*D*_*H*_ ratio and no regions of high [*H*]. See S1 Movie legend for additional details.
(MOV)

S11 Movie Simulation 11 in 2D of the two-molecule HB model. This simulation generates the Fig 5A panel with a high *D*_*B*_/*D*_*H*_ ratio. See S1 Movie legend for additional details.
(MOV)

S12 Movie Simulation 12 of the HBPM model in 3D with high *D*_*H*_ and medium *D*_*B*_. See S1 Movie legend for additional details.
(MOV)

S13 Movie Simulation 13 of the HBPM model in 3D with medium *D*_*H*_ and low *D*_*B*_. See S1 Movie legend for additional details.
(MOV)

S14 Movie Simulation 14 of the HBPM model in 3D with low *D*_*H*_ and medium *D*_*B*_. See S1 Movie legend for additional details.
(MOV)

S15 Movie Simulation 15 of eustele. Fig 3P. See S1 Movie legend for additional details.
(MOV)

S16 Movie Simulation 16 of eustele with central SVBs. Fig 3Q. See S1 Movie legend for additional details.
(MOV)

S17 Movie Simulation 17 of siphonostele with central SVBs. Fig 3R. See S1 Movie legend for additional details.
(MOV)

S18 Movie Simulation 18 of atactostele. Fig 3V. See S1 Movie legend for additional details.
(MOV)

S19 Movie Simulation 19 with apical production of A and basipetal transport by *P*. *D*_*H*_>0. Note that the movie switches view from the cross section at the apex of the cylinder to a cross section mid-way through the cylinder at time point 16 to highlight the differences in patterning at the apex and center of the cylinder. See S1 Movie legend for additional details.
(MOV)

S1 Fig Apical production of *A* with basipetal transport by *P* in the augmented *HBPM* model. ***D***_***H***_ **= 0.** Snapshot of transverse section through simulated stem. Regions of high [*H*] are narrow and scattered but differ from typical vascular patterns.
(PNG)

## References

[1] Carroll SB. Evo-devo and an expanding evolutionary synthesis: A genetic theory of morphological evolution. Cell. 2008 pp. 25–36. 10.1016/j.cell.2008.06.030 18614008

[2] Niklas KJ. Evolutionary biology of plants University of Chicago Press; 1997.

[3] Carlquist S. More woodiness/less woodiness: Evolutionary avenues, ontogenetic mechanisms. Int J Plant Sci. 2013;174: 964–991. 10.1086/670400

[4] Ibañes M, Fàbregas N, Chory J, Caño-Delgado AI. Brassinosteroid signaling and auxin transport are required to establish the periodic pattern of *Arabidopsis* shoot vascular bundles. Proc Natl Acad Sci U S A. 2009;106: 13630–5. 10.1073/pnas.0906416106 19666540 PMC2717112

[5] Benítez M, Hejátko J. Dynamics of Cell-Fate Determination and patterning in the vascular bundles of *Arabidopsis thaliana*. PLoS One. 2013;8 10.1371/journal.pone.0063108 23723973 PMC3664626

[6] Cartenì F, Giannino F, Schweingruber FH, Mazzoleni S. Modelling the development and arrangement of the primary vascular structure in plants. Ann Bot. 2014;114: 619–627. 10.1093/aob/mcu074 24799440 PMC4156123

[7] Muraro D, Mellor N, Pound MP, Help H, Lucas M, Chopard J, et al Integration of hormonal signaling networks and mobile microRNAs is required for vascular patterning in *Arabidopsis* roots. Proc Natl Acad Sci U S A. 2014;111: 857–62. 10.1073/pnas.1221766111 24381155 PMC3896157

[8] Ramachandran P, Carlsbecker A, Etchells JP, Turner S. Class III HD-ZIPs govern vascular cell fate: An HD view on patterning and differentiation. J Exp Bot. 2016;68: 55–69. 10.1093/jxb/erw370 27794018

[9] Fàbregas N, Formosa-Jordan P, Ibañes M, Caño-Delgado AI. Experimental and theoretical methods to approach the study of vascular patterning in the plant shoot Humana Press, New York, NY; 2017 pp. 3–19. 10.1007/978-1-4939-6722-3_1 28050824

[10] Feller C, Farcot E, Mazza C. Self-organization of plant vascular systems: Claims and counter-claims about the flux-based auxin transport model. PLoS One. 2015;10: 1–18. 10.1371/journal.pone.0118238 25734327 PMC4348546

[11] De Rybel B, Mähönen AP, Helariutta Y, Weijers D. Plant vascular development: from early specification to differentiation. Nat Rev Mol Cell Biol. Nature Publishing Group; 2015;17: 30–40. 10.1038/nrm.2015.6 26580717

[12] Hearn DJ. Perennial growth, form and architecture of angiosperm trees In: Groover AT, Cronk QCB, editors. Comparative and evolutionary genomics of angiosperm trees. Springer, New York, NY; 2016 pp. 179–204. 10.1007/7397_2016_25

[13] Headon DJ, Painter KJ. Stippling the skin: Generation of anatomical periodicity by reaction-diffusion mechanisms. Math Model Nat Phenom. 2009;4: 83–102. 10.1051/mmnp/20094402

[14] Chang C, Wu P, Baker RE, Maini PK, Alibardi L, Chuong C-M. Reptile scale paradigm: Evo-Devo, pattern formation and regeneration. Int J Dev Biol. 2009;53: 813–826. 10.1387/ijdb.072556cc 19557687 PMC2874329

[15] Widelitz RB, Xin Jiang T, Shen T, Shen J, Chuong C. Molecular biology of feather morphogenesis: A testable model for evo-devo research. J Exp Zool B Mol Dev Evol. 2003; 298(1): 109–122 10.1002/jez.b.29 12949772 PMC4382008

[16] Bard JBL. A model for generating aspects of zebra and other mammalian coat patterns. J Theor Biol. 1981;93: 363–385. 10.1016/0022-5193(81)90109-0 7334825

[17] Murray JD. On pattern formation mechanisms for lepidopteran wing patterns and mammalian coat markings. Philos Trans R Soc Lond B Biol Sci. 1981;295: 473–496. 10.1098/rstb.1981.0155 6117906

[18] Nijhout HF, Maini PK, Madzvamuse A, Wathen AJ, Sekimura T. Pigmentation pattern formation in butterflies: Experiments and models. Comptes Rendus—Biol. 2003;326: 717–727. 10.1016/j.crvi.2003.08.004 14608692

[19] Meinhardt H. The algorithmic beauty of sea shells 4th ed. New York: Springer-Verlag; 2009.

[20] Metz HC, Manceau M, Hoekstra HE. Turing patterns: how the fish got its spots. Pigment Cell Melanoma Res. 2011;24: 12–14. 10.1111/j.1755-148X.2010.00814.x 21118391

[21] Reinhardt D, Pesce E-R, Stieger P, Mandel T, Baltensperger K, Bennett M, et al Regulation of phyllotaxis by polar auxin transport. Nature. 2003;426: 255–260. 10.1038/nature02081 14628043

[22] Smith RS, Guyomarc’h S, Mandel T, Reinhardt D, Kuhlemeier C, Prusinkiewicz P. A plausible model of phyllotaxis. Proc Natl Acad Sci U S A. 2006;103: 1301–1306. 10.1073/pnas.0510457103 16432192 PMC1345713

[23] Schellmann S, Schnittger A, Kirik V, Wada T, Okada K, Beerman A, et al TRIPTYCHON and CAPRICE mediate lateral inhibition during trichome and root hair patterning in *Arabidopsis*. EMBO J. 2002;21: 5036–5046. 10.1093/emboj/cdf524 12356720 PMC129046

[24] Benítez M, Monk NAM, Alvarez-Buylla ER. Epidermal patterning in *Arabidopsis*: models make a difference. J. Exp. Zool. 2011; 316:241–253. 10.1002/jez.b.21398 21259417

[25] Turing AM. The Chemical Basis of Morphogenesis. Philos Trans R Soc Lond B Biol Sci. 1952;237: 37–72. 10.1098/rstb.2014.0218 PMC4360114 25750229

[26] Munteanu A, Solé R V. Neutrality and robustness in evo-devo: Emergence of lateral inhibition. PLoS Comput Biol. 2008;4 10.1371/journal.pcbi.1000226 19023404 PMC2577890

[27] Holwill PJA. Occurrence of medullary bundles in the apple shoot. Nature. 1950;165: 156–157.

[28] Carlquist S. Comparative wood anatomy: Systematics, ecological, and evolutionary aspects of dicotyledon wood. 2nd ed. Berlin, Heidelberg: Springer Berlin Heidelberg; 2001;

[29] Mauseth JD. The Structure of photosynthetic succulent stems in plants other than cacti. Int J Plant Sci. 2004;165: 1–9.

[30] Hearn DJ. Developmental patterns in anatomy are shared among separate evolutionary origins of stem succulent and storage root-bearing growth habits in *Adenia* (Passifloraceae). Am J Bot. 2009;96: 1941–1956. 10.3732/ajb.0800203 21622314

[31] Hearn DJ. Dissection of evolutionary networks to assess their role in the evolution of robustness, function, and diversification. Evolution (N Y). 2013;67: 2273–2283. 10.1111/evo.12120 23888850

[32] Mauseth JD. Medullary bundles and the evolution of cacti. Am J Bot. 1993;80: 928–932.

[33] Mauseth JD, Sajeva M. Cortical bundles in the persistent, photosynthetic stems of cacti. Ann Bot. 1992;70: 317–324. 10.1093/oxfordjournals.aob.a088480

[34] Janse van Vuuren DR. ‘n morfologiese studie van die stingels van die houtagtige verteenwoordigers van die genus *Adenia* Forsk. in suid-afrika. Universiteit van Pretoria 1970.

[35] Hearn DJ. Adenia (Passifloraceae) and its adaptive radiation: Phylogeny and growth form diversification. Syst Bot. 2006;31: 805–821. 10.1600/036364406779695933

[36] Hearn DJ, O’Brien P, Poulsen TM. Comparative transcriptomics reveals shared gene expression changes during independent evolutionary origins of stem and hypocotyl/root tubers in *Brassica* (Brassicaceae). PLoS ONE 2018; 13(6): e0197166 10.1371/journal.pone.0197166 29856865 PMC5983522

[37] Wei J, Winter M. Stationary multiple spots for reaction-diffusion systems. J Math Biol. 2008;57: 53–89. 10.1007/s00285-007-0146-y 18058100

[38] Gray P, Scott SK. Autocatalytic reactions in the isothermal, continuous stirred tank reactor. Chem Eng Sci. 1984;39: 1087–1097. 10.1016/0009-2509(84)87017-7

[39] Schnakenberg J. Simple chemical reaction systems with limit cycle behaviour. J Theor Biol. 1979;81: 389–400. 10.1016/0022-5193(79)90042-0 537379

[40] Raser JM, O’Shea EK. Noise in gene expression: Origins, consequences, and control. Science (80-). 2005;309: 2010–2013. 10.1126/science.1105891 16179466 PMC1360161

[41] Schlogl F. Chemical reaction models for non-equilibrium phase transitions. Zeitschrift fur Phys. 1972;253: 147–161. 10.1007/BF01379769

[42] Gillespie DT. Exact stochastic simulation of coupled chemical reactions. J Phys Chem. 1977;81: 2340–2361. 10.1021/j100540a008

[43] Slepoy A, Thompson AP, Plimpton SJ. A constant-time kinetic Monte Carlo algorithm for simulation of large biochemical reaction networks. J Chem Phys. 2008;128: 0–8. 10.1063/1.2919546 18513044

[44] Ramaswamy R, Sbalzarini IF. Exact on-lattice stochastic reaction-diffusion simulations using partial-propensity methods. J Chem Phys. 2011;135 10.1063/1.3666988 22225140

[45] Hutton T, Munafo R, Trevorrow A, Rokicki T, Wills D. Ready ver. 0.8, a cross-platform implementation of various reaction-diffusion systems [Internet]. 2016 Available: https://github.com/GollyGang/ready

[46] Wardlaw CW. Morphogenesis in plants London: Methuen; 1952.

[47] Hearn DJ, Poulsen T, Spicer R. The evolution of growth forms with expanded root and shoot parenchymatous storage is correlated across the eudicots. Source Int J Plant Sci. 2013;174: 1049–1061. 10.1086/671745

[48] Torrey JG. On the determination of vascular pattern formation during tissue differentiation in excised pea roots. Am. J. Bot. 1955; 42: 183–198

[49] Torrey JG. Auxin control of vascular pattern formation in regenerating pea root meristems grown in vitro. Am. J. Bot. 1957; 44: 859–870

[50] Newman SA, Bhat R. Dynamical patterning modules: a "pattern language" for development and evolution of multicellular form. Int. J. Dev. Biol. 2009; 53: 693–705 10.1387/ijdb.072481sn 19378259

[51] Granero MI, Porati a, Zanacca D. A bifurcation analysis of pattern formation in a diffusion governed morphogenetic field. J Math Biol. 1977;4: 21–7. 845509 10.1007/BF00276349

[52] Misbah C. Complex dynamics and morphogenesis: an introduction to nonlinear science. Springer; 2017.

[53] Nenninger A, Mastroianni G, Mullineaux CW. Size dependence of protein diffusion in the cytoplasm of *Escherichia coli*. J Bacteriol. 2010;192: 4535–4540. 10.1128/JB.00284-10 20581203 PMC2937421

[54] Lipman DJ, Souvorov A, Koonin E V., Panchenko AR, Tatusova TA. The relationship of protein conservation and sequence length. BMC Evol Biol. 2002;2: 20 10.1186/1471-2148-2-20 12410938 PMC137605

[55] Baima S, Possenti M, Matteucci A, Wisman E, Altamura MM, Ruberti I, et al The *Arabidopsis* ATHB-8 HD-ZIP protein acts as a differentiation-promoting transcription factor of the vascular meristems. Plant Physiol. 2001;126: 643–655. 10.1104/pp.126.2.643 11402194 PMC111156

[56] Emery JF, Floyd SK, Alvarez J, Eshed Y, Hawker NP, Izhaki A, et al Radial patterning of *Arabidopsis* shoots by *Class III HD-ZIP* and *KANADI* genes. Curr Biol. 2003;13: 1768–1774. 10.1016/j.cub.2003.09.035 14561401

[57] Prigge MJ, Otsuga D, Alonso JM, Ecker JR, Drews GN, Clark SE. Class III homeodomain-leucine zipper gene family members have overlapping, antagonistic, and distinct roles in *Arabidopsis* development. Plant Cell. 2005;17: 61–76. 10.1105/tpc.104.026161 15598805 PMC544490

[58] Zhong R, Ye ZH. Amphivasal vascular bundle 1, a gain-of-function mutation of the *IFL1*/*REV* gene, is associated with alterations in the polarity of leaves, stems and carpels. Plant Cell Physiol. 2004;45: 369–385. 10.1093/pcp/pch051 15111711

[59] Byrne ME. Shoot meristem function and leaf polarity: The role of *Class III HD-ZIP* genes. PLoS Genet. 2006;2: 0785–0790. 10.1371/journal.pgen.0020089 16846251 PMC1484593

[60] Ilegems M, Douet V, Meylan-Bettex M, Uyttewaal M, Brand L, Bowman JL, et al Interplay of auxin, KANADI and Class III HD-ZIP transcription factors in vascular tissue formation. Development. 2010;137: 975–984. 10.1242/dev.047662 20179097

[61] Du J, Miura E, Robischon M, Martinez C, Groover A. The *Populus* Class III HD ZIP transcription factor *POPCORONA* affects cell differentiation during secondary growth of woody stems. PLoS One. 2011;6 10.1371/journal.pone.0017458 21386988 PMC3046250

[62] Donner TJ, Sherr I, Scarpella E. Regulation of preprocambial cell state acquisition by auxin signaling in *Arabidopsis* leaves. Development. 2009;136: 3235–3246. 10.1242/dev.037028 19710171

[63] Caño-Delgado A, Yin Y, Yu C, Vafeados D, Mora-García S, Cheng J-C, et al BRL1 and BRL3 are novel brassinosteroid receptors that function in vascular differentiation in *Arabidopsis*. Development. The Company of Biologists Ltd; 2004;131: 5341–5351. 10.1242/dev.01403 15486337

[64] Liu L, Zinkgraf M, Petzold HE, Beers EP, Filkov V, Groover A. The *Populus* ARBORKNOX1 homeodomain transcription factor regulates woody growth through binding to evolutionarily conserved target genes of diverse function. New Phytol. 2015;205: 682–694. 10.1111/nph.13151 25377848

[65] McConnell JR, Emery J, Eshed Y, Bao N, Bowman J, Barton MK. Role of PHABULOSA and PHAVOLUTA in determining radial patterning in shoots. Nature. 2001;411: 709–713. 10.1038/35079635 11395776

[66] Sessa G, Morelli G, Ruberti I. The Athb-1 and -2 HD-Zip domains homodimerize forming complexes of different DNA binding specificities. EMBO J. 1993;12: 3507–17. 8253077 10.1002/j.1460-2075.1993.tb06025.x PMC413627

[67] Ramachandran R, Carlsbecker A, Etchells JP. Class III HD-ZIPs govern vascular cell fate: an HD view on patterning and differentiation. J. Exp. Bot. 2017; 68: 55–69. 10.1093/jxb/erw370 27794018

[68] Sachs T. Polarity and the induction of organized vascular tissues. Ann Bot. 1969;33: 263–275. 10.1093/oxfordjournals.aob.a084281

[69] Sachs T. The control of the patterned differentiation of vascular tissues. Adv Bot Res. 1981;9: 151–262. 10.1016/S0065-2296(08)60351-1

[70] Sachs T. Cell polarity and tissue patterning in plants. Development. 1991;113: 83–93.

[71] Kleine-Vehn J, Ding Z, Jones AR, Tasaka M, Morita MT, Friml J. Gravity-induced PIN transcytosis for polarization of auxin fluxes in gravity-sensing root cells. Proc Natl Acad Sci U S A. 2010;107: 22344–22349. 10.1073/pnas.1013145107 21135243 PMC3009804

[72] Friml J, Wiśniewska J, Benková E, Mendgen K, Palme K. Lateral relocation of auxin efflux regulator PIN3 mediates tropism in *Arabidopsis*. Nature. 2002;415: 806–809. 10.1038/415806a 11845211

[73] Bennett T, Hines G, van Rongen M, Waldie T, Sawchuk MG, Scarpella E, et al Connective auxin transport in the shoot facilitates communication between shoot apices. PLoS Biol 2016; 14(4): e1002446 10.1371/journal.pbio.1002446 27119525 PMC4847802

[74] Scarpella E, Marcos D, Friml J, Berleth T, Scarpella E, Marcos D, et al Control of leaf vascular patterning by polar auxin transport. 2006; 1015–1027. 10.1101/gad.1402406 16618807 PMC1472298

[75] Scarpella E, Meijer AH. Pattern formation in the vascular system of monocot and dicot plant species. New Phytologist. 2004 10.1111/j.1469-8137.2004.01191.x 33873557

[76] Zhou GK, Kubo M, Zhong R, Demura T, Ye ZH. Overexpression of miR165 affects apical meristem formation, organ polarity establishment and vascular development in *Arabidopsis*. Plant Cell Physiol. 2007;48: 391–404. 10.1093/pcp/pcm008 17237362

[77] Furuta KM, Hellmann E, Helariutta Y. Molecular control of cell specification and cell differentiation during procambial development. Annu Rev Plant Biol. 2014; 1–32. 10.1146/annurev-arplant-050213-040306 24579995

[78] Crawford KM, Zambryski PC. Non-targeted and targeted protein movement through plasmodesmata in leaves in different developmental and physiological states. Plant Physiol. 2001;125: 1802–1812. 10.1104/pp.125.4.1802 11299360 PMC88836

