# Supplementary material for: Turing-like mechanism in a stochastic reaction-diffusion model recreates three dimensional vascular patterning of plant stems
Source: PLoS One. 2019 Jul 24;14(7):e0219055. doi: 10.1371/journal.pone.0219055 (PMC6715405; doi:10.1371/journal.pone.0219055)
Supplement: S1 Appendix — (DOCX) [file pone.0219055.s001.docx]

**S1 Appendix**

**Algorithmic details of the stochastic reaction-diffusion simulation**

The Gillespie algorithm [1] provides an exact stochastic simulation of reaction systems based on roots in Monte Carlo Markov process theory of Doob [2,3]. As an exact simulation, a sampled reaction trajectory represents a solution of an associated stochastic master equation. To formalize, the reaction system consists of a set *R_i_*, of reactant concentrations (expressed as numbers of reactant molecules in a reaction volume), a set, *X_i_*, of reactions, a set of reaction rates *k_i_* (one per reaction *X*_i_), and a set of propensity functions *F*(*X_i_*, *k_i_*, *V*). Probabilities of a reaction occurring are based on the law of mass action in a well-mixed, physically homogeneous reaction volume. Under the law of mass action, the probability that a particular reaction occurs in a given time interval is proportional to the number of ways the reactants can interact (which is expressed as a product of the reactant concentrations) times an intrinsic rate constant divided by the reaction volume. This calculation defines the propensity for the reaction. Taking the limit as the time interval becomes infinitesimally small leads to exponentially distributed times between reaction events. Reaction events are conditionally independent so that the time to the next reaction occurs exponentially with rate equal to the sum of all propensities of all the reactions. The probability that reaction *X_i_* occurs at a sampled time, τ, is Pr(Reaction=*X_i_* | τ) = *F*(*X_i_*, *k_i_*) / Σ*_j_ F*(*X_j_*, *k_j_*). Once a reaction occurs, reactant concentrations and reactant propensities are updated based on the number of reactants consumed and the number of products produced during the reaction. Until the next reaction, these concentrations and propensities do not change, so the probabilities of reactions do not change until the next reaction event.

Turing [4] recognized that a stable equilibrium state in a well-mixed system can become unstable in a (possibly) heterogeneous system in which reactants diffuse in space. Such processes can be simulated by breaking the total reaction volume into a set of equal-volume regions, or “voxels”, in which conditions are assumed to be homogeneous and reactants can diffuse between neighboring voxels. Diffusion may be considered a type of ‘reaction’ and incorporated into the stochastic simulation algorithm (SSA) above. Like other reaction events, diffusion events have a propensity equal to the diffusion rate of the reactant times the number of reactant molecules in the voxel divided by the volume of the voxel (e.g., [5–7]). In the simplest model of diffusion, a reactant’s diffusion rate is the same in every voxel, and a molecule diffuses into any of the neighboring, equal-volume voxels with equal probability. Thus, diffusion is modeled as a conservative process in which the total concentration of molecules does not change during diffusion events.

Collectively, the simulation of reaction and diffusion events constitutes a reaction-diffusion system. To fully specify a reaction-diffusion system, initial reactant concentrations and boundary conditions are defined. Boundary conditions define the geometry of the total reaction volume and define what happens to reactants that diffuse in voxels at the periphery of the reaction volume. When diffusion conserves the total number of molecules, reactants reflect against the periphery, so they never leave the reaction volume.

Additionally, constraints that define when and where reactions occur can be specified so that some reactions are restricted to particular regions of the reaction volume at particular times. A reaction constraint is a Boolean 0 or 1 value for each voxel for each reaction, *I*(*X_i_*, *voxel_j_*) ; a 0 Boolean value for a voxel indicates the reaction cannot occur in the voxel. A reaction’s propensity for voxel *j* is then *F*(*X_i_*, *k_i_*, *I_i,j_*) = *F*(*X_i_*, *k_i_*) ** I*(*X_i_*, *voxel_j_*).

Substantial improvements have been made to Gillespie’s algorithm that dramatically increased computational efficiency. In Gillespie’s original SSA, the time to carry out the algorithm scaled linearly with the number of reactions. Improvements provided by Slepoy et al. [8] reduced the time complexity to constant in the number of reactions in weakly coupled reaction networks [9], meaning that simulation time was independent of the number of reactions in the reaction-diffusion system. This innovation made it feasible to simulate enormous reaction-diffusion systems with tens of millions of reaction events in seconds

In a reaction diffusion system with a grid lattice of 100 X 100 X 100 equal-volume voxels, two reactions, and two reactants, the total number of reactions (including diffusion events) to track during the simulation is 100*100*100*6*2*2 or 24 million reactions. So, even relatively small-reaction diffusion systems require the simulation of tens of millions of reactions. In equal-volume voxels, the probability calculations are independent of the voxel volume, and the reaction event times are proportional to the reciprocal of the volume.

Below, the algorithmic details are provided:

Definitions:

1. Define reactants *R_i_*, reactions, *X_i_*, reaction rates, *k_i_*, reactant diffusion rates, *D_i_*.
2. Define the grid lattice with dimensions *X*, *Y*, and *Z*, and the volume of each lattice voxel to define total reaction volume, *V*, as the sum of the volumes of all voxels. The current simulation used a cubical lattice of equal-volume voxels to approximate a cylindrical solid.
3. Define the reaction dependency graph. This graph describes which reactants’ concentrations, and which reaction propensities, need updating when a particular reaction occurs. Gibson and Bruck [10] provide details.

Initialization:

1. Initialize a uniform random number generator.
2. Initialize the grid lattice, boundary conditions, reactant concentrations for each voxel (i.e., [*R_i,voxel(j)_*]) , spatial (and temporal) constraints on reactions.
3. Calculate reaction propensities based on initial reactant concentrations for each reaction in each voxel; a total propensity for each reaction, *T_Xi_*, equal to the sum of propensities for reaction *X_i_* in all voxels of the lattice; a total global reaction propensity, *T_X_*, equal to the sum of all reaction propensities, *T_Xi_*.
4. Calculate the total diffusion propensities, *T_Ri_,* for each reactant based on the total number of molecules of each reactant and the reactant diffusion rate; calculate the total global diffusion propensity, *T_D_*, equal to the sum of all *T_Ri_*.
5. Calculate the total global propensity, *G*, as the sum *T_X_* + *T_D_*
6. Initialize bin contents for composition-rejection sampling ([8] provides details)
7. Initialize time and number of iterations to 0

Run:

1. Sample the time to the next reaction, *τ*, according to an exponential distribution with parameter *G*, and update the time and iteration variables. *τ* ~ *exp*(*G*)
2. Determine whether a diffusion event or a reaction event occurs. Pr(Event = Diffusion) = *T_D_* / *G*
3. If Event is Diffusion
   1. Determine which reactant diffuses using a discrete distribution sampling algorithm such as binary roulette wheel sampling. Pr(Reactant = *R*_i_) = *T_Ri_* / *T_D_*
   2. Determine from which voxel the diffusion event takes place using composition-rejection sampling. Pr(*voxel* = *j*) = [*R_i,voxel(j)_*]/ [*R_iTotal_*]
   3. Determine to which voxel the reactant diffuses. Under passive diffusion, each neighboring voxel has equal probability of being diffused into.

If Event is Reaction

1. Determine which reaction occurs using binary roulette wheel sampling. P(Reaction = *X_i_*) = *T_Xi_*/ *T_X_*
2. Determine in which voxel the reaction event takes place using composition-rejection sampling. Pr(*voxel*=*j*) = *F*(*X_i_*, *k_i_*, *I_i,j_*) / *T_Xi_*
3. Update reactant concentrations and propensities following the reaction Event based on the reaction dependency graph.
4. Repeat steps 1. – 5. until a termination criterion, such as a predefined time, criterion for stationarity, or number of iterations, is reached.

**References**

1. Gillesple DT. Exact Stochastic Simulation of couple chemical reactions. J Phys Chem. 1977;81: 2340–2361. doi:10.1021/j100540a008

2. Doob JL. Topics in the theory of Markoff chains. Trans Am Math Soc. 1942;52: 37–37. doi:10.1090/S0002-9947-1942-0006633-7

3. Doob JL. Markoff Chains--Denumerable Case. Trans Am Math Soc. 1945;58: 455–473.

4. Turing AM. The Chemical Basis of Morphogenesis. Philos Trans R Soc Lond B Biol Sci. 1952;237: 37–72.

5. Erban R, Chapman J, Maini P. A practical guide to stochastic simulations of reaction-diffusion processes. arXiv Prepr arXiv07041908. 2007; 35.

6. Engblom S, Ferm L, Hellander A, Lötstedt P. Simulation of stochastic reaction-diffusion processes on unstructured meshes. SIAM J Sci …. 2008; 1774–1797. doi:10.1137/080721388

7. Ramaswamy R, Sbalzarini IF. Exact on-lattice stochastic reaction-diffusion simulations using partial-propensity methods. J Chem Phys. 2011;135. doi:10.1063/1.3666988

8. Slepoy A, Thompson AP, Plimpton SJ. A constant-time kinetic Monte Carlo algorithm for simulation of large biochemical reaction networks. J Chem Phys. 2008;128: 0–8. doi:10.1063/1.2919546

9. Ramaswamy R, Sbalzarini IF. A partial-propensity variant of the composition-rejection stochastic simulation algorithm for chemical reaction networks. J Chem Phys. 2010;132. doi:10.1063/1.3297948

10. Gibson MA, Bruck J. Efficient Exact Stochastic Simulation of Chemical Systems with Many Species and Many Channels. J Phys Chem A. 2000;104: 1876–1889. doi:10.1021/jp993732q
