# Supplementary material for: Turing-like mechanism in a stochastic reaction-diffusion model recreates three dimensional vascular patterning of plant stems
Source: PLoS One. 2019 Jul 24;14(7):e0219055. doi: 10.1371/journal.pone.0219055 (PMC6715405; doi:10.1371/journal.pone.0219055)
Supplement: S1 Program — This file is a ZIP archive file of multiple files. In addition to the executable jar file, the ZIP file includes a tutorial, source code in the .jar file, and java library files (lib directory) required to run the reaction-diffusion program. (ZIP) [file pone.0219055.s004.zip › S1Program/reactiondiffusionProgramDocumentation.pdf]

Stochastic reaction diffusion software documentation

Author: David J. Hearn

Version: 0.1

This software is open-source and is available for free. If used for other projects, please include the citation of the associated paper with which this software was bundled.

I. WHAT IT IS

THIS SOFTWARE SIMULATES A SYSTEM OF REACTIONS IN SPACE AND TIME USING A STOCHASTIC REACTION-DIFFUSION FRAMEWORK. A "REACTION" IS ANY INTERACTION BETWEEN SYSTEM COMPONENTS (MOLECULES, SPECIES, INDIVIDUALS IN POPULATIONS, ETC.) THAT RESULTS IN CHANGES IN THE NUMBERS OR POSITIONS OF THE COMPONENTS.

THE SOFTWARE USES THE GILLESPIE STOCHASTIC SIMULATION ALGORITHM WITH COMPOSITION-REJECTION SAMPLING.

II. IMPLEMENTATION

THE SOFTWARE IS WRITTEN IN JAVA AND COMES WITH A PRE-COMPILED JAR FILE THAT CAN BE RUN ON ANY OS WITH JAVA7.0 OR LATER INSTALLED. THE FULL SOURCE CODE IS ALSO INCLUDED IN THE JAR FILE FOR FURTHER SOFTWARE DEVELOPMENT AND COMPILATION.

III. INSTALLATION

DOWNLOAD BOTH THE JAR FILE AND THE lib/ DIRECTORY THAT CONTAINS THREE ADDITIONAL JAR FILES NEEDED TO RUN THE PROGRAM

IV. RUNNING THE SOFTWARE

A. FROM THE WINDOWS ENVIRONMENT, DOUBLE CLICK THE JAR FILE

B. FROM THE COMMAND LINE:

CHANGE DIRECTORY TO THE DIRECTORY WITH THE JAR FILE, AND TYPE, E.G.:

```
java -d64 -mx2000m -cp ./lib -jar ReactionDiffusion.jar
```

This assumes java is in your PATH and is java7.0 or higher

V. USER INTERFACE

A. THE USER INTERFACE PROVIDES A MECHANISM TO SET UP, RUN, GRAPH, AND ANIMATE YOUR SIMULATIONS OF SYSTEM OF REACTIONS.

B. CURRENTLY, THE SIMULATION AND THE GRAPHICAL USER INTERFACE MUST BE RUN TOGETHER.

VI. MODEL SET UP (IN THE INITIALIZE MENU)

A. SELECT GRID

1. SET THE SPATIAL CONFIGURATION YOUR SIMULATION. THE PROGRAM WILL CREATE A LATTICE OF EQUAL-VOLUME CUBES BETWEEN WHICH MOLECULES FREELY DIFFUSE.

2. SPATIAL UNITS ARE A CONSTANT MULTIPLE OF THE NUMBER OF X, Y, AND Z CELLS.

B. SELECT REACTIONS

1. ONCE YOU HAVE SET THE GRID DIMENSIONS, YOU CAN THEN ENTER THE SYSTEM OF REACTIONS YOU WISH TO MODEL.

A) THE NUMBER OF REACTANTS IS CURRENTLY LIMITED TO 52 – THE UPPER AND LOWER CASE LETTERS.

B) THE NUMBER OF REACTIONS IS LIMITED BY THE MEMORY AND DISC SPACE OF YOUR COMPUTER.

2. EACH REACTION GOES ON A SEPARATE LINE

3. EACH REACTANT IN A REACTION IS REPRESENTED AS A SINGLE (CASE-SENSITIVE) LETTER.

4. EACH REACTION HAS ONE OF THE FOLLOWING FORMATS (NO SPACE BETWEEN LETTERS AND SYMBOLS):

A) <REACTANT LETTER>[+<REACTANT LETTER>+...+<REACTANT LETTER>]=<REACTANT LETTER>[+<REACTANT LETTER>+...+<REACTANT LETTER>]:<REACTION RATE>

B) 0=<REACTANT LETTER>[+<REACTANT LETTER>+...+<REACTANT LETTER>]:<RATE OF PRODUCTION>

C) <REACTANT LETTER>[+<REACTANT LETTER>+…+<REACTANT LETTER>]=0:<RATE OF DEGRADATION>

D) <REACTANT LETTER>UPDOWN<REACTANT LETTER>:<TRANSPORT RATE IN LONGITUDINAL DIRECTION>

E) <REACTANT LETTER>SIDEWAYS<REACTANT LETTER>:<TRANSPORT RATE IN LATERAL DIRECTION>

F) <REACTANT LETTER>DOWN<REACTANT LETTER>:<TRANSPORT RATE IN DOWNWARD DIRECTION>

5. THE EQUALS SIGN REPRESENTS 'YIELD', AND IT CAN BE WRITTEN '->' INSTEAD OF '='.

6. SEVERAL EXAMPLE REACTION SYSTEMS ARE PROVIDED WITH THE JAR FILE AND CAN BE ACCESSED BY RUNNING THE PROGRAM AND SELECTING AN EXAMPLE UNDER THE FILE…EXAMPLE REACTIONS MENU. TYPE SHIFT+P TO SEE A BASIC DESCRIPTION OF THE MODEL AND SOME SUGGESTIONS FOR VISUALIZATION. CLICK THE "REACTION RATES" BUTTON TO SEE THE LIST OF REACTIONS AND THEIR ASSOCIATED RATES.

SOME BASIC REACTION SYSTEMS INCLUDE:

(1) EXPONENTIAL GROWTH:

0=A:1

(2) LOGISTIC GROWTH

0=A:50

A+A=0:0.1

C. SELECT INITIAL CONDITIONS

1. ONCE YOU HAVE SET YOUR REACTIONS, YOU CAN SPECIFY THE INITIAL REACTANT CONCENTRATIONS

2. SELECT THE REACTANT WHOSE INITIAL CONCENTRATIONS YOU WANT TO SET

3. IF THE SAME INITIAL CONCENTRATION IS WANTED THROUGHOUT THE REACTION VOLUME, SELECT UNIFORM

4. IF THE INITIAL CONCENTRATION IS LIMITED TO A CYLINDRICAL SHELL, SELECT CYLINDER

A) PUT THE RADIUS (IN µM) OF THE OUTERMOST WALL OF THE SHELL.

B) PUT THE WIDTH (IN µM) OF THE SHELL.

5. SET THE INITIAL NUMBER OF MOLECULES PER GRID CELL

6. THE DEFAULT NUMBER IS 0 MOLECULES PER GRID CELL

D. SELECT DIFFUSION

1. ONCE YOU HAVE SET THE REACTANT INITIAL CONDITIONS, YOU CAN SET THE REACTANT DIFFUSION RATES

2. SETTING DIFFUSION RATES USES THE FOLLOWING FORMAT:

A) <DIFFUSION RATE>:<LOWEST CONCENTRATION FOR COLOR PALETTE>,<HIGHEST CONCENTRATION FOR COLOR PALETTE>

B) THE COLOR PALETTE (SELECTABLE FROM MENU OPTIONS…PALETTE…) IS THE COLORING USED TO VISUALIZE THE CONCENTRATIONS OF THE REACTANTS IN EACH GRID CELL

E. SELECT REACTION CONSTRAINTS

1. ONCE YOU HAVE SET DIFFUSION RATES, YOU CAN RESTRICT WHERE THE REACTIONS WILL OCCUR.

2. IF YOU WANT TO CONSTRAIN A REACTION TO A SET OF GRID CELLS, THIS MENU OPTION ALLOWS YOU TO DO THIS WITH LIMITED CONTROL.

3. YOU CAN CONSTRAIN THE REACTION TO A CYLINDRICAL SHELL (C.F. INITIAL CONDITIONS), THE TOP LAYER, OR A DOT AT THE TOP OF THE REACTION VOLUME, OR LEAVE IT UNIFORM (DEFAULT).

4. YOU CAN ALSO EDIT THE SAVED REACTION FILE AND MANUALLY CONSTRAIN THE REACTION TO ANY GEOMETRY BY SETTING THE CONTSTRAINT VALUES IN THE DESIRED GRID CELLS TO 0. A 0 MEANS THE REACTION WILL NOT OCCUR IN THE GRID CELL.

VII. SET OTHER SIMULATION PARAMETERS

A. SELECT MENU OPTIONS…SET PARAMETERS…

1. THIS WILL BRING UP A TABBED PANE

2. THE TABS ARE:

A) OUTPUT

(1) SET THE OUTPUT DIRECTORY WHERE OUTPUT FILES WILL BE SAVED

(2) THE OUTPUT DIRECTORY WILL BE CREATED IN THE USER HOME FOLDER .reaction_diffusion FOLDER

B) SAMPLING

(1) DO NOT ALTER UNLESS YOU KNOW WHAT YOU ARE DOING.

C) ANIMATION: WHILE THE SIMULATION IS RUNNING, IMAGES OF THE REACTANT'S CONCENTATIONS ARE AUTOMATICALLY SAVED AT A USER-SPECIFIED RATE. THESE IMAGES CAN BE VIEWED IN RAPID SUCCESSION TO MAKE AN ANIMATION OF THE SIMULATION.

(1) SET UP THE INFORMATION FOR MAKING AN ANIMATION (AKA MOVIE), INCLUDING:

(A) THE MINIMUM NUMBER OF IMAGES NEEDED TO MAKE AN ANIMATION,

(B) THE FREQUENCY THAT IMAGES ARE SAVED,

(C) THE MOVIE SIZE (LEAVE AT -1 FOR DEFAULT MOVIE SIZE)

(D) THE FRAME RATE (THIS DESCRIBES THE NUMBER OF IMAGES USED PER SECOND OF MOVIE TIME),

(E) THE IMAGE SKIP INTERVAL SPECIFIES THE HOW MANY OF THE AUTOMATICALLY-SAVED IMAGES TO SKIP BETWEEN IMAGES USED IN THE MOVIE

(F) THE "FIRST IMAGES NUMBER TO SKIP" SPECIFIES HOW MANY INITIAL IMAGES SHOULD BE SKIPPED AND NOT INCLUDED IN THE MOVIE

(G) CLICK THE MAKE IMAGES WITH ALL REACTANTS CHECKBOX TO HAVE THE IMAGES INCLUDE GRAPHICS FOR ALL THE REACTANTS

(I) IN 3D SIMULATIONS, YOU CAN ALSO INCLUDE

(A) THE LONGITUDINAL SECTION

(B) THE TIME SERIES OF CONCENTRATIONS

(C) THE NUMBER OF COLUMNS (ONE PER REACTANT) IN THE OUTPUT IMAGES

D) DISPLAY

(1) SET THE MINIMUM AND MAXIMUM CONCENTRATION VALUES FOR DISPLAY

(2) LEAVE AT -1 IF YOU WANT THE PROGRAM TO DETERMINE THIS AUTOMATICALLY

E) SIMULATION

(1) THIS TAB PANE KEEPS TRACK OF THE SIMULATION TIME AND NUMBER OF ITERATIONS

VIII. MAKING AN ANIMATION (AKA MOVIE) OF YOUR SIMULATION

1. SELECT OPTIONS...RENDER IMAGES TO TOGGLE AUTOMATIC SAVING OF IMAGES. DEFAULT IS ON

2. SET THE SIMULATION PARAMETERS...ANIMATION TAB PANE PARAMETERS, AS DESCRIBED EARLIER.

3. SELECT A REACTANT TO DISPLAY

4. SET THE Z-STACK POSITION (3D SIMULATIONS ONLY)

5. SET THE NUMBER OF REACTION INTERACTIONS BEFORE THE DISPLAY IS UPDATED

6. CLICK RUN.

7. ONCE THE MINIMUM NUMBER OF IMAGES FOR A MOVIE IN SAVED, THE “VIEW ANIMATION” BUTTON BECOMES ENABLED.

A) CLICK THE VIEW ANIMATION BUTTON

B) WATCH THE ANIMATION WITH THE ANIMATION RUN, PAUSE, STEP, AND RESET BUTTONS.

C) WHEN YOU ARE SATISFIED WITH THE ANIMATION GRAPHICS, SELECT SAVE MOVIE

(1) THE MOVIE WILL BE SAVED AUTOMATICALLY AND THE FILE NAME WILL BE PROVIDED

D) IF YOU ARE NOT SATISFIED WITH THE ANIMATION TRY THE FOLLOWING:

(1) ADJUST THE ANIMATION PARAMETERS

(2) RESTART THE SIMULATION

(3) PICK A DIFFERENT COLOR PALETTE: OPTIONS…PALETTE

IX. SAVING AND OPENING SIMULATION DATA

A. AUTOSAVING

1. THE FULL REACTION SYSTEM INFORMATION CAN BE SAVED AUTOMATICALLY BY TOGGLING FILE…AUTOSAVE (ON BY DEFAULT)

2. YOU CAN AUTOSAVE IMAGES OF THE CONCENTRATION PANEL (OPTIONS…RENDER IMAGES)

3. YOU CAN AUTOSAVE ALL CONCENTRATION VALUES AT EACH POINT IN TIME (OPTIONS…SAVE DATA)

B. SAVING

1. THE REACTION SYSTEM AND SIMULATION STATE CAN BE SAVED: FILE…SAVE…MODEL

2. YOU CAN ALSO SAVE INDIVIDUAL IMAGES AND CONCENTRATION DATA FOR A GIVEN TIME POINT.

C. OPENING

PREVIOUSLY SAVED MODEL FILES CAN BE REOPENED AND RUN FROM THE LAST SAVE POINT. THESE FILES HAVE THE .rdm EXTENSION

X. ADVANCED

1. THE FILES ARE SAVED TO A SUB-FOLDER WITHIN THE .reaction_diffusion DIRECTORY IN THE USER'S HOME DIRECTORY. SEE VII.A.2.A) ABOVE FOR DETAILS.

2. WHEN THE PROGRAM IS STARTED, A NEW FOLDER FOR THE CURRENT SESSION WILL AUTOMATICALLY BE CREATED IN THE .reaction_diffusion FOLDER.

3. THE MODEL FILE (ENDING WITH .rdm) SPECIFIES THE SIMULATION PARAMETERS, REACTIONS, DIFFUSION RATES, INITIAL CONDITIONS, CURRENT CONDITIONS, AND REACTION CONSTRAINTS.

A) THE INITIAL CONDITIONS, CURRENT CONDITIONS, AND REACTION CONSTRAINTS CAN BE MANUALLY SET IN THIS FILE.

B) REACTION CONSTRAINTS ARE EITHER '1', IN WHICH CASE THE REACTION CAN OCCUR IN THE GRID CELL, OR '0' IN WHICH CASE THE REACTION IS NOT ALLOWED TO OCCUR IN THE GRID CELL.

C) THE INITIAL CONDITIONS, CURRENT CONDITIONS, AND REACTION CONSTRAINTS ARE LISTED AS A SEQUENCE OF NUMBERS. KNOWING THE X.Y. AND Z DIMENSIONS, AND THE INDEX IN THE ARRAY, YOU CAN FIGURE OUT THE COORDINATES OF THE CELL WITHIN THE ARRAY:

(1) Z = (INT) INDEX / (DIM_Y * DIM_X);

(2) Y = (INT) ((INDEX % (DIM_Y * DIM_X)) / DIM_X);

(3) X = ((INDEX % (DIM_Y * DIM_X)) % DIM_X) / 1;

D) OTHER PARAMETERS CAN BE SET IN THE .rdm FILE RATHER THAN THROUGH THE GRAPHICAL USER INTERFACE USING A TEXT EDITOR.

EACH ENTRY IN THE .rdm FILE HAS THE FORMAT

```
<PARAMETER NAME>=<PARAMETER VALUE>
```

E) IF YOU HAVE A .rdm MODEL FILE, YOU CAN START THE PROGRAM BY READING THE MODEL FILE:

```
java -d64 -mx2000m -cp ./lib -jar ReactionDiffusion.jar <Model file>
```

XI. OTHER FEATURES

1. SOME EXAMPLE MODELS ARE INCLUDED UNDER FILE…EXAMPLE MODELS

2. YOU CAN CLICK THE CONCENTRATION PANEL TO GRAPH THE TIME SERIES OF THE DISPLAYED REACTANT'S CONCENTRATIONS IN THE CELL THAT WAS CLICKED.

3. YOU CAN GRAPH THE TOTAL CONCENTRATION BY CLICKING THE RADIO BUTTON UNDER THE TIME SERIES GRAPH

4. A LOG PROVIDES SOME BASIC INFORMATION, ALTHOUGH IT IS NOT FULLY IMPLEMENTED

XII. WARNINGS

A. GENERAL

1. THE AUTHOR OF THE SOFTWARE CANNOT GUARANTEE THE ACCURACY OF THE SIMULATION RESULTS.

2. YOU MUST COMPLETELY REINITIALIZE THE MODEL IF YOU WISH TO CHANGE ANY MODEL PARAMETERS.

3. CLICKING ON DIFFERENT POINTS OF THE CONCENTRATION PANEL WILL ERASE PRIOR CONCENTRATION DATA FOR THE PREVIOUSLY CLICKED POINT OF THE CONCENTRATION PANEL

4. SOME SIMULATIONS CAN TAKE A LONG TIME!! CODE IS NOT PARALLELIZED.

B. KNOWN BUGS / IMPLEMENTATION ISSUES

1. IF AN ERROR '45149: COULD NOT PROCESS EXAMPLE MODEL' OCCURS, CLOSE AND RESTART THE PROGRAM. RESELECT THE EXAMPLE MODEL.

2. THE SIMULATION CANNOT CURRENTLY BE RUN WITHOUT THE GRAPHICAL USER INTERFACE. THIS LIMITATION MAKES IT DIFFICULT TO RUN MULTIPLE MODELS SIMULTANEOUSLY.

3. UNITS OF MEASUREMENT ARE NOT IMPLEMENTED.

   (1) THE TIMES ARE NOT IN SECONDS, BUT A CONSTANT MULTIPLE OF SECONDS

   (2) THE UNITS OF VOLUME ARE A CONSTANT MULTIPLE OF THE SIMULATED VOLUMES.
