# Supplementary figures and images for: Turing-like mechanism in a stochastic reaction-diffusion model recreates three dimensional vascular patterning of plant stems
Source: PLoS One. 2019 Jul 24;14(7):e0219055. doi: 10.1371/journal.pone.0219055 (PMC6715405; doi:10.1371/journal.pone.0219055)

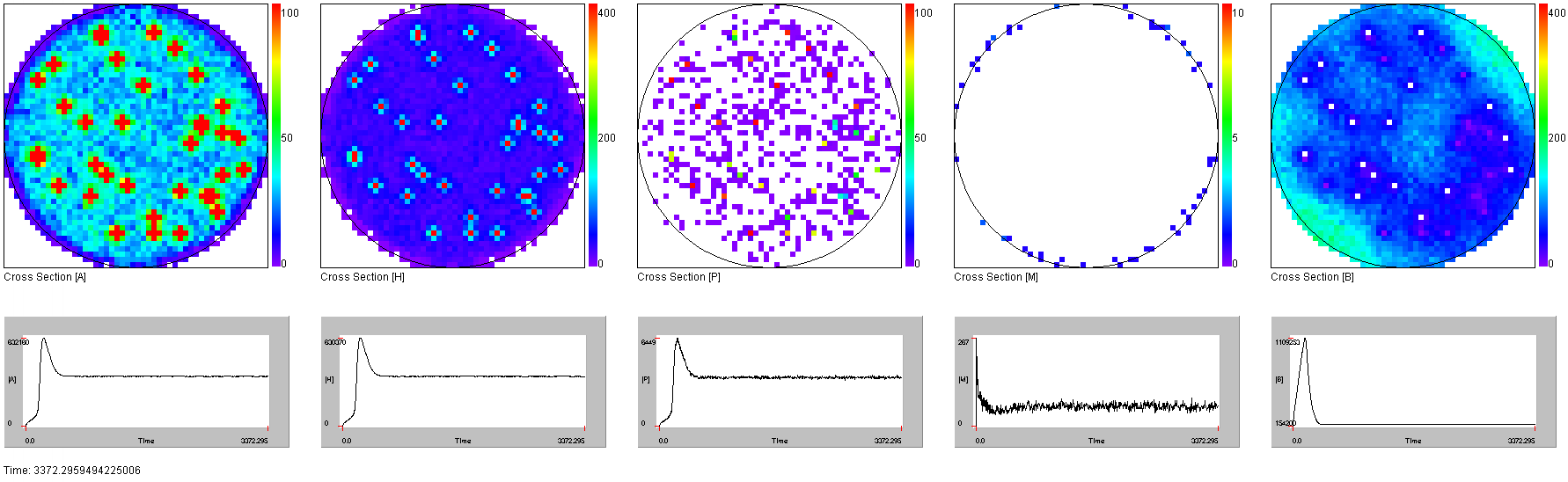

Supplement: S1 Fig — DH = 0. Snapshot of transverse section through simulated stem. Regions of high [H] are narrow and scattered but differ from typical vascular patterns. (PNG) [file pone.0219055.s026.PNG]
